# COMP-Angiopoietin-1 accelerates muscle regeneration through N-cadherin activation

**DOI:** 10.1038/s41598-018-30513-7

**Published:** 2018-08-17

**Authors:** Seock-Won Youn, Hyun-Chae Lee, Sae-Won Lee, Jaewon Lee, Hyunduk Jang, Eun Ju Lee, Hyo-Soo Kim

**Affiliations:** 1 Center of Cell- & Bio-Therapy for Heart, Diabetes, and Cancer, Seoul National University Hospital, Seoul, Korea; 2 Department of Molecular Medicine and Biopharmaceutical Sciences, Graduate School of Convergence Science and Technology, Seoul National University, Seoul, Korea

## Abstract

Angiopoietin-1 modulates vascular stability via Tie2 on endothelial cells. In our previous study, we also showed it acts as an inhibitor of cardiomyocyte death. However, it remains poorly understood how Ang1 regulates myogenesis during muscle regeneration. Here we found that COMP-Ang1 (cAng1) enhances muscle regeneration through N-cadherin activation. Muscle fiber regeneration after limb muscle damage by ischemic injury was enhanced with cAng1 treatment. Mechanistically cAng1 directly bound to N-cadherin on the myoblast surface in a Ca^2+^ dependent manner. The interaction enhanced N-cadherin activation via N-cadherin/p120-catenin complex formation, which in turn activated p38MAPK (but not AKT or ERK) and myogenin expression (but not myoD) as well as increasing myogenin^+^ cells *in/ex vivo*. After transplantation of GFP-expressing myoblasts (GFP-MB), we showed an increased generation of GFP^+^ myotubes with adenovirus cAng1 (Adv-cAng1) injection. Adv-cAng1, however, could not stimulate myotube formation in N-cadherin-depleted GFP-MB. Taken together, this study uncovers the mechanism of how cAng1 promotes myoblast differentiation and muscle regeneration through the N-cadherin/p120-catenin/p38MAPK/myogenin axis.

## Introduction

Skeletal muscle maintains its function by continuous regeneration of new muscle fibers^1,2^. After normal or pathologic muscle damage, myoblasts proliferate, differentiate, and fuse with each other to form multinucleated myotubes or fuse with myofibers, and reconstitute the contractile function of skeletal muscle^2^. It is important to understand the cellular and molecular mechanisms underlying skeletal muscle regeneration because it may lead to the development of cell therapies to treat diseases such as skeletal muscle degeneration. The regeneration process of skeletal muscle^3,4^ is tightly controlled by the muscle vasculature and angiogenic factors including basic fibroblast growth factor, insulin-like growth factor 1, and vascular endothelial growth factor.

Angiopoietin-1 (Ang1) is a well-known angiogenic factor and COMP-Angiopoietin-1 (COMP-Ang1; cAng1) is a derivative of native Ang1 that is more potent than native Ang1^5^. Ang1 is usually produced from pericytes and smooth muscle cells and is known to bind to the Tie2 receptor, which is an endothelial receptor-tyrosine kinase^6–8^. The main function of Ang1 is to maintain vessel constructs during vessel differentiation in embryogenesis^9,10^, and to augment non-leaky vessel formation through the stabilization of adherens junctions or through tight junction formation in endothelial cells^11–13^. cAng1 suppresses apoptosis of endothelial cells in hypoxic conditions and increases the adhesive capacity of circulating progenitor cells^14,15^. Ang1 also enhances survival of skeletal and cardiac myocytes and promotes myoblast proliferation and differentiation *in vitro*^8,16–19^. We previously reported that cAng1 increased cardiomyocyte survival after hypoxic-reoxygenation damage by activating integrin β1 signaling^11^. However, it remains poorly understood how Ang1 regulates myogenesis during muscle regeneration.

In this study, we found that cAng1 promotes myogenesis by binding to N-cadherin, but not integrin β1, in a Ca^2+^-dependent manner. cAng1 binding activated the N-cadherin/p120-catenin/p38MAPK/myogenin axis. Additionally, Adv-cAng1 increased regenerating muscle fiber formation after ischemic injury and myotube formation in transplantation myoblasts. However, the increase was not observed in N-cadherin-‘depleted’ myoblasts. Our data demonstrates the importance of N-cadherin in cAng1-mediated muscle regeneration.

## Results

### cAng1 accelerates muscle regeneration after ischemic injury

**Figure 1:**
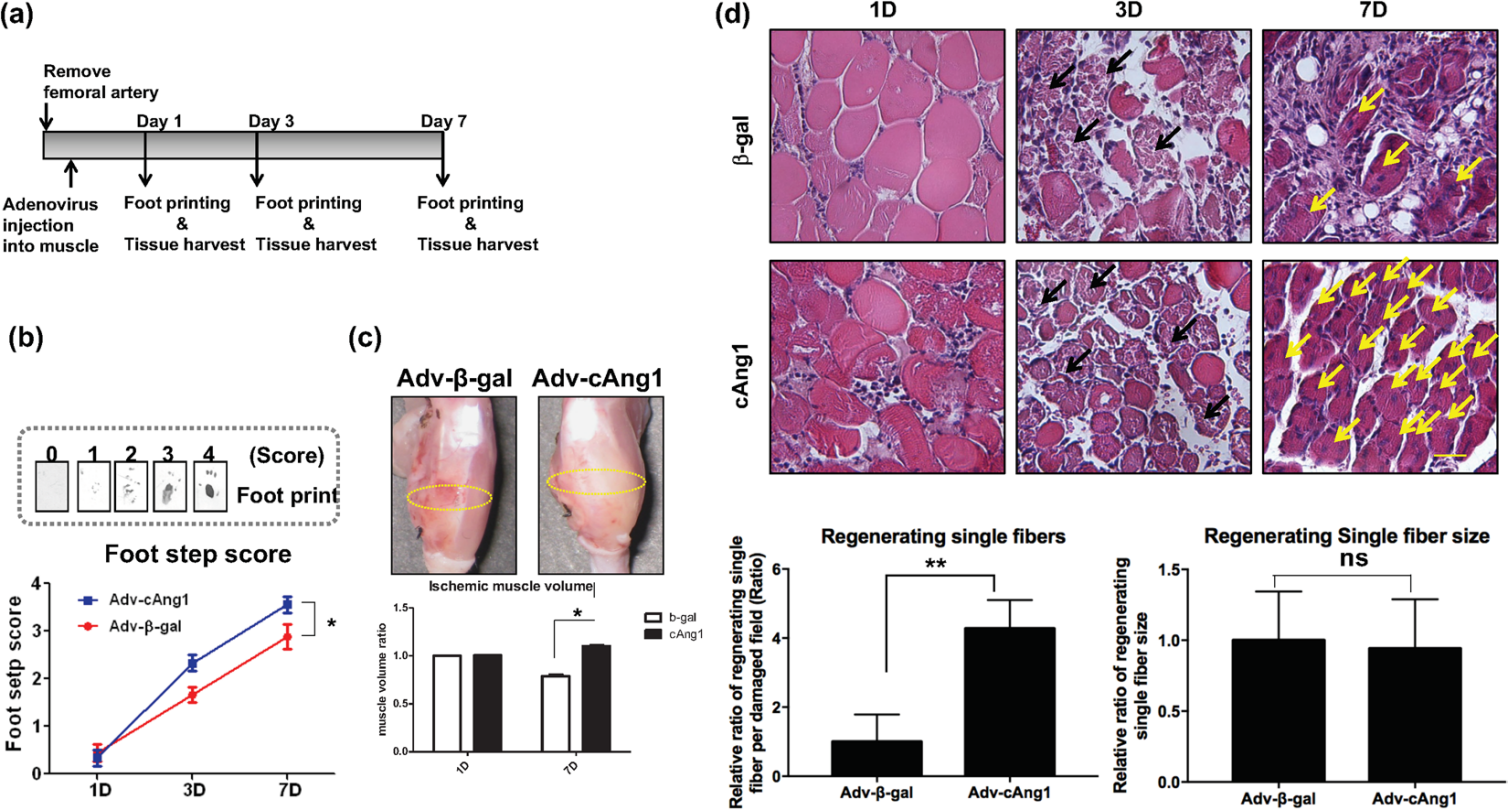
cAng1 accelerates muscle regeneration after ischemic injury. (**a**) Timeline of the hindlimb ischemic model. (**b**) The foot recovery scores. Non-printing foot was indicated as a “0” score, only prints of touched foot was indicated as a “1” score, prints of the toe was indicated as a “2” score, prints of toes and sole of the foot was indicated as a “3” score, and prints of a fully recovered foot was indicated as a “4” score. The foot step scores of Adv-cAng1 mice (n = 5) were significantly higher than that of Adv-β-gal mice (n = 5), Mean value ± SEM, n = 5, *p < 0.05. (**c**) The measurement of muscle volume after ischemic injury. The muscle volume ratio was calculated by ischemic/normal gastrocnemius muscle at 1 and 7 days. Mean value ± SEM, n = 3, *p < 0.05. (**d**) Histology of ischemia damaged muscle on time dependent manner. Adv-cAng1 injection dramatically increased regenerating muscle fibers (yellow arrows) after ischemic injury at 7 days. Scale bar = 100 μm, Magnification, x200. The regenerating single fiber numbers were counted in damaged field area with image J, normalized by Adv-β-gal. The regenerating single fiber size on cross section was measured by image J and was normalized by Adv-β-gal.

Our previous studies have shown the effects of cAng1 on angiogenesis and the recruitment of vascular progenitor cells into areas injured by ischemia^11,14,15^. In this study, we focused on the effects of cAng1 on muscle regeneration. We examined *in vivo* whether cAng1 contributes to the regeneration of damaged muscle using the mouse hindlimb ischemic model (Fig. 1), which has been used to study muscle regeneration^20,21^. We infected adenovirus-expressing-cAng1 (Adv-cAng1) or adenovirus-β-galactosidase (Adv-β-gal) into skeletal muscle after ischemic injury (Fig. 1a). Adv-cAng1 enhanced the foot recovery score (Fig. 1b) and increased muscle volume (Fig. 1c) more than Adv-β-gal. Histological analysis of Adv-cAng1 injected samples showed dramatically increased muscle fiber regeneration (arrows) compared to Adv-β-gal injected samples but the size of the regenerating fibers was similar between both groups at day 7 after ischemic injury (Fig. 1d and supplementary Fig. 1). These data indicate that cAng1 could enhance muscle regeneration after ischemic injury.

### cAng1 induces the differentiation of C2C12 myoblasts to myotubes

**Figure 2:**
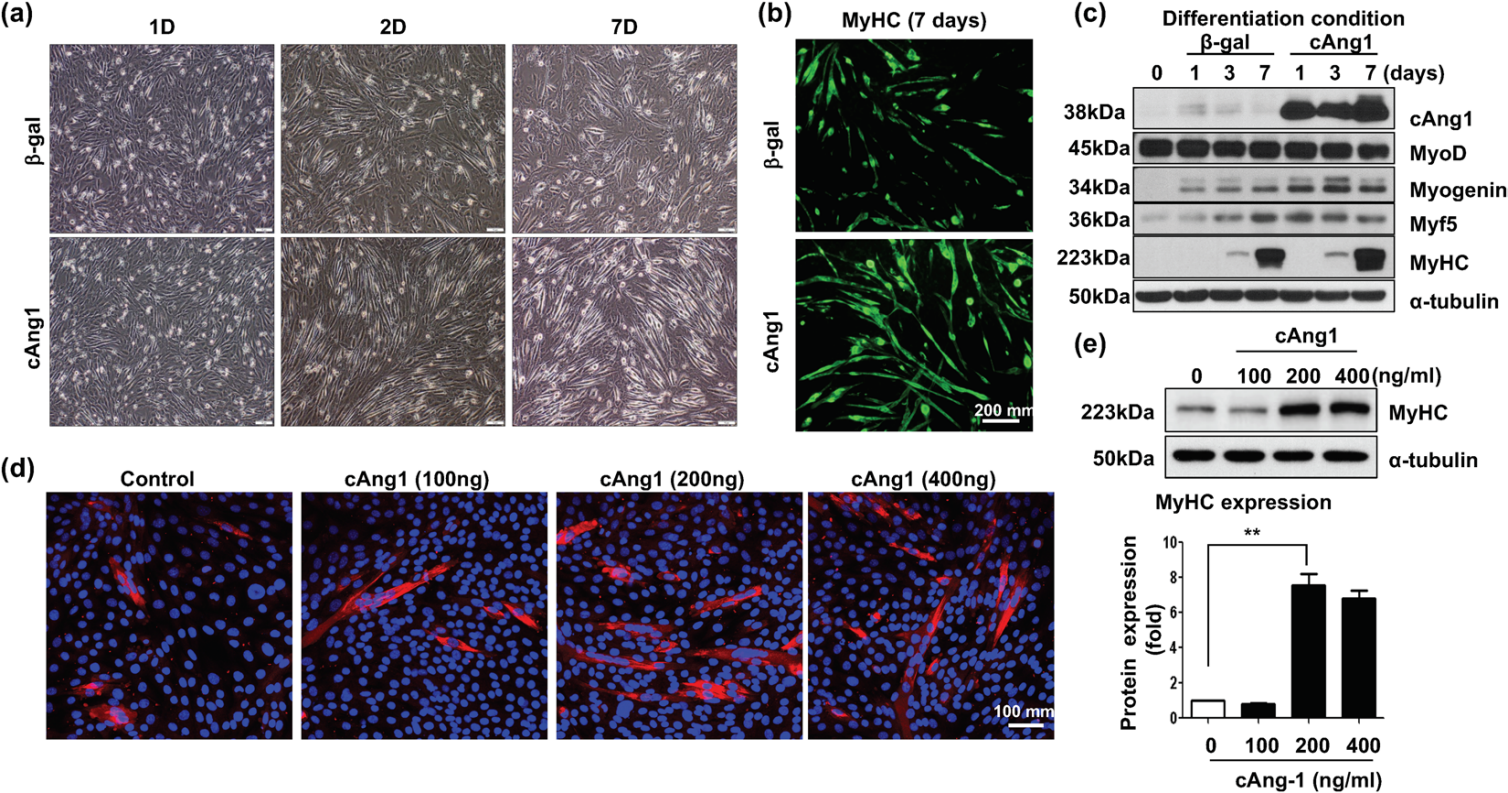
cAng1 is a stimulator of myoblast differentiation. (**a**) Bright field images of C2C12 myoblast differentiation. After adenovirus infection, C2C12 were treated with differentiation media for 7 days. Scale bar = 200 μm. Magnification, x100. (**b**) Immunofluorescent staining of MyHC. Myoblast differentiation was evaluated with MyHC stain (green) at 7 days. Scale bar = 200 μm. Magnification, x100. (**c**) Immunoblot to confirm myogenesis in C2C12 myoblast. After adenovirus infection, C2C12 were harvested and analyzed myogenic markers such MyoD, myogenin, Myf5, and MyHC. cAng1 was detected by anti-Flag. α-tubulin was used to internal control. (**d**) The myotubes (MyHC, red) were increased by cAng1 in a dose dependent manner at 7 day differentiation. Cell nucleus was indicated on blue by DAPI staining. Scale bar = 100 μm. Magnification, x200. (**e**) Immunoblotting for MyHC after cAng1 treatment. The recombinant cAng1 concentration (200 and 400 ng/ml) enhanced MyHC expression in myoblasts after 3 days. Quantification graph for MyHC (fold). (Mean value ± SEM, n = 3, **P < 0.001).

We assessed the effects of cAng1 on myoblast differentiation by infecting Adv-β-gal and Adv-cAng1 into C2C12 myoblasts under myotube differentiation conditions. After 7 days in differentiation media, myoblasts were able to make myotubes in both groups. However, Adv-cAng1 infected myoblasts had more myotube formation compared with Adv-β-gal infected myoblasts in a time dependent manner (Fig. 2a). Moreover, Adv-cAng1 enhanced myosin heavy chain (MyHC) positive myotube generation at differentiation day 7 (Fig. 2b). Myogenesis is regulated by myogenic transcriptional factor induction and myogenic gene expression in a time dependent manner^22^. Therefore, we investigated the expression of myogenic markers and MyHC at different time points. Myogenic marker expression such as Myf5 and myogenin was increased after 1 day of differentiation and MyHC was increased after 7 days of differentiation as reported^22^. Interestingly, Adv-cAng1 increased myogenic markers and MyHC expression compared to Adv-β-gal (Fig. 2c). We also investigated the effects of recombinant cAng1 (cAng1) and native Ang1 (nAng1) under differentiation conditions to analyze out-side-in signaling. We found that cAng1 significantly increased both myotube formation and MyHC expression at 200–400 ng/ml at differentiation day 7 (Fig. 2d,e). nAng1 also induced myotube formation, while the myogenic effect of cAng1 was much stronger than nAng1 (Supplementary Fig. 2). Since we found that 200 ng/ml of cAng1 was the most effective concentration to induce myotube formation and MyHC expression, we used this concentration of cAng1 for subsequent *in vitro* studies.

### cAng1 induces myogenin expression through p38MAPK

**Figure 3:**
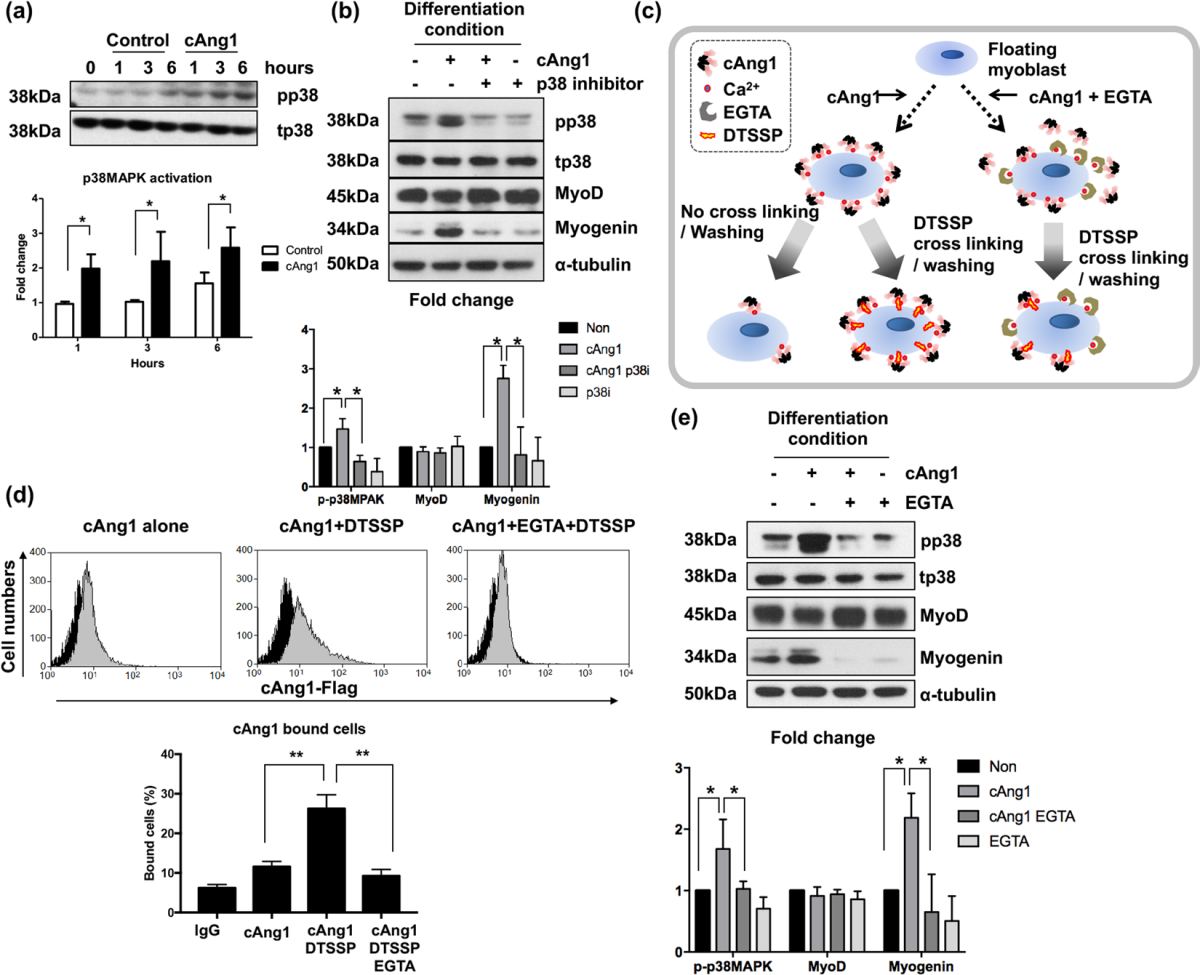
cAng1 enhances myogenin expression through p38MAPK in a Ca^2+^ dependent manner. (**a**) Immunoblotting to confirm signaling molecules by cAng1 (200 ng/ml). Myoblasts were exposed to differentiation media and treated with cAng1. p38MAPK was activated by cAng1. Quantitative graph was made from three independent experiments (Mean value ± SEM, *P < 0.05). (**b**) cAng1 increased the myogenin expression at 1 day after differentiation, which was suppressed by treatment with the p38MAPK inhibitor (SB239063, 10 μM). MyoD expression was not changed by cAng1 or the p38MAPK inhibitor. p38MAPK activation was confirmed at 3 hours (Mean value ± SEM, n = 3, *P < 0.05). (**c**) Schematic diagram of surface binding experiment. Detached myoblasts were incubated with cAng1 and underwent cross-linking by DTSSP. EGTA was used as a Ca^2+^ ion chelating agent. The binding amount of cAng1 on the surface of myoblasts was evaluated with FACS. (**d**) The analysis of cAng1 binding capacity on the myoblast surface. The histogram indicated anti-Flag without cAng1 (black) as control and with cAng1 (gray). Without DTSSP, cAng1 could not stably bind on the myoblast surface. The stable binding amount of cAng1 was increased by DTSSP but reduced by EGTA (Mean value ± SEM, n = 4, **P < 0.01). (**e**) In the immunoblot analysis, cAng1 increased the p38MAPK phosphorylation at 3 hours and myogenin expression at 1 day, which was suppressed by EGTA. The quantification graph of myogenin expression ratio was shown (Mean value ± SEM, n = 3, *P < 0.05 and **P < 0.01).

We then investigated the downstream signal pathways activated by cAng1 during myoblast differentiation (Fig. 3). p38MAPK, but not AKT or ERK, was activated by cAng1 (Fig. 3a and Supplementary Fig. 3). We next assessed the expression of key myogenic transcription factors, MyoD and myogenin^23^. The expression of myogenin, but not MyoD, was significantly increased by cAng1 and completely suppressed by the p38MAPK inhibitor (SB239063) even in the presence of cAng1 (Fig. 3b). Moreover, the effects of cAng1, such as p38MAPK activation and myogenin expression, were suppressed by Tie2 transfection in differentiation conditions although AKT was activated (Supplementary Fig. 4). These results indicate that p38MAPK is one of the major downstream pathways required for upregulation of myogenin expression by cAng1.

### cAng1 directly binds to the myoblast surface in a Ca^2+^-dependent manner

Both nAng1 and cAng1 contain a fibrinogen like domain on their C-terminal end^5^. The fibrinogen like domain contains numerous Ca^2+^ binding sites similar to fibrinogen. It has also been reported that Ang1 binding to myoblasts is dependent on Ca^2+^ ^16^. Therefore, we examined whether cAng1 binds to the myoblast surface in a Ca^2+^-dependent manner under differentiation conditions. Myoblasts were incubated with cAng1 in suspension to increase the chance of contact between the cells and cAng1. After incubation, cells were fixed with DTSSP, a non-permeable protein-protein cross linker^24^, and the cAng1-binding capacity on the myoblast surface was analyzed with FACS (Fig. 3c). cAng1 binding on the myoblast surface was low without DTSSP, as the binding of cAng1 on myoblasts is transient. Importantly, cAng1 binding on the myoblast surface was increased by the addition of DTSSP while it was significantly reduced by the addition of EGTA, a high affinity Ca^2+^ chelator^16,25^ (Fig. 3d). We also confirmed that cAng1-induced myogenesis signaling was also Ca^2+^ dependent. We found that both p38MAPK activation and myogenin expression increased by cAng1 were suppressed by EGTA treatment (Fig. 3e). These data suggest that cAng1 binds to the myoblast surface in a Ca^2+^-dependent manner, thereby activating p38MAPK, which is involved in myogenin expression.

### N-cadherin acts as a receptor for cAng1 on the myoblast surface during myogenesis

**Figure 4:**
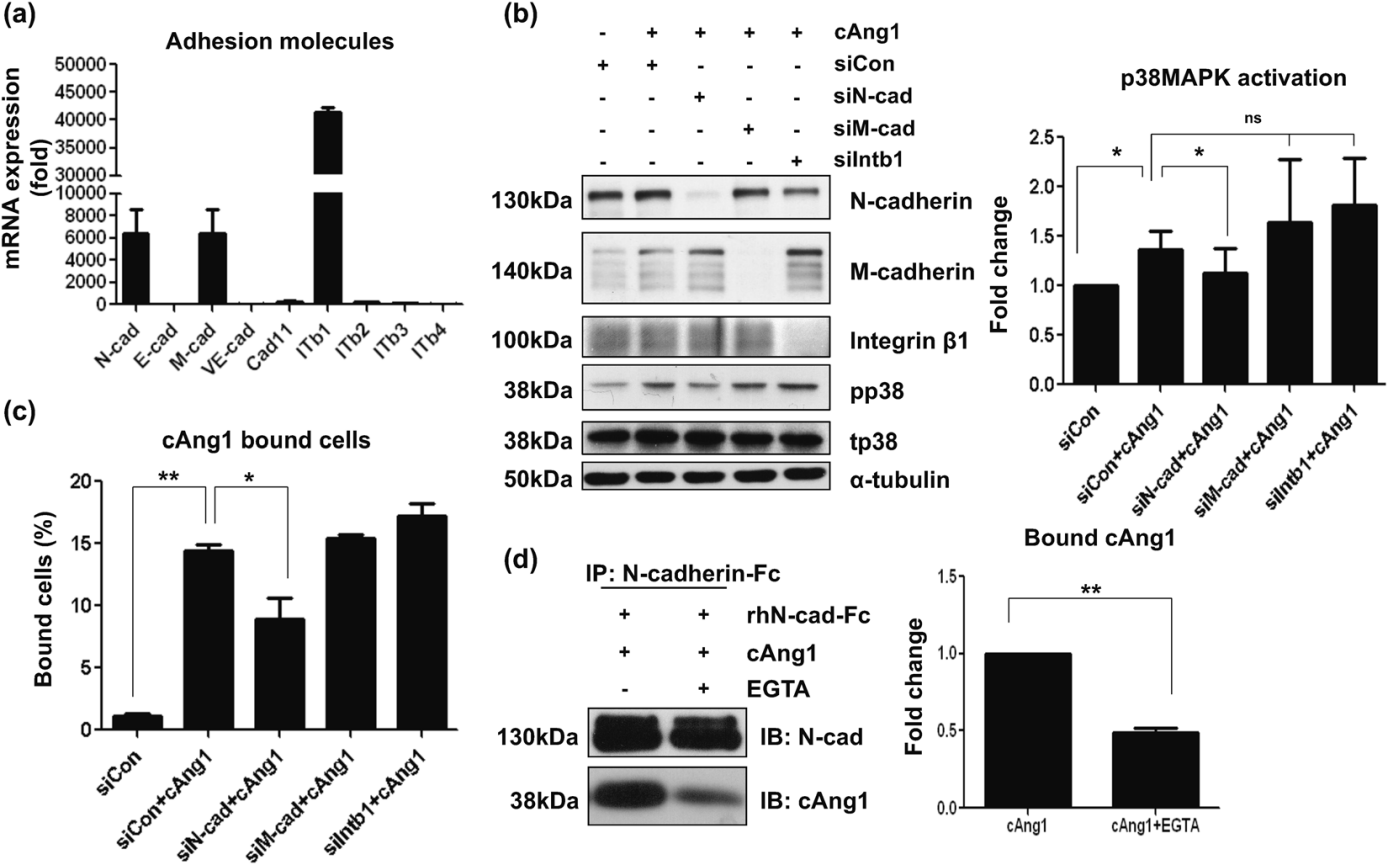
cAng1 directly binds to N-cadherin on the myoblast surface. (**a**) mRNA levels of adhesion molecules in myoblasts. N-cadherin (N-cad), M-cadherin (M-cad) and integrin β1 (ITb1) were abundantly expressed in myoblasts. The relative value was based on E-cadherin (E-cad) expression. ITb2, integrin β2; ITb3, integrin β3; ITb4, integrin β4. (**b**) Western blotting after knockdown with siRNA. Each sample was transfected with siRNA against N-cadherin (siN-cad), M-cadherin (siM-cad), and Integrin β1 (siIntb1). Knockdown of N-cadherin significantly inhibited p38MAPK activation in the presence of cAng1 at 3 hours. The knockdown of M-cadherin or integrin β1 did not significantly influence cAng1 effects on p38MAPK activation, respectively. Quantification graph of the p38MAPK phosphorylation ratio was assessed by densitometry of different immunoblots (Mean value ± SEM, n = 4, *P < 0.05 and n.s. = none signification). (**c**) cAng1 binding assay to target adhesion molecules. The surface binding of cAng1 on myoblasts was significantly reduced by siN-cadherin, but not by siM-cadherin or siIntegrin β1. The graph of cAng1 binding capacity was assessed with different FACS analyses (Mean value ± SEM, n = 3, *P < 0.05 and **P < 0.01). (**d**) The *in vitro* binding assay for N-cadherin with cAng1. The interaction between human N-cadherin-Fc and cAng1-Flag was confirmed with anti-Flag and anti-N-cadherin antibody. The pull-downed cAng1 by anti-N-cadherin-antibody was detected with anti-Flag antibody, which was significantly reduced by EGTA treatment (Mean value ± SEM, n = 3, **P < 0.01).

Since myoblasts do not express Tie2, an endothelial specific receptor for Ang1^26^, we sought to determine which surface molecules on the myoblast can act as a cAng1 receptor. We focused on the cadherin and integrin families of receptors because the structure of cadherins and integrins have numerous Ca^2+^ binding domains and their activity is regulated by Ca^2+^ ^16,27^. We first evaluated the mRNA expression of cadherin and integrin family members. N-cadherin, M-cadherin, and integrin β1 were expressed in myoblasts (Fig. 4a). Therefore, to investigate the effects of N-cadherin, M-cadherin, and integrin β1 on p38MAPK signaling, we depleted the expression of N-cadherin, M-cadherin, and integrin β1 with specific siRNA (Fig. 4b). As shown in Fig. 4b, the expression of each surface molecule was specifically knocked down by the respective siRNA, but not by control non-silencing siRNA (siCon). Knockdown of N-cadherin significantly inhibited p38MAPK activation in the presence of cAng1, but not M-cadherin or integrin β1 (Fig. 4b). Ang1 was previously reported to bind to integrin β1 on myoblasts in the presence of Ca^2+^ in an adhesion assay using Ang1 coated plates^16^. To evaluate cAng1 binding to the myoblast surface under differentiation conditions, we knocked down N-cadherin, M-cadherin, and integrin β1 in myoblasts and assessed surface binding with FACS. The surface binding of cAng1 on myoblasts was significantly reduced by depletion of N-cadherin, but not by depletion of M-cadherin or integrin β1 (Fig. 4c). We also confirmed that N-cadherin expression was not reduced on the myoblast surface during trypsinization (Supplementary Fig. 5). This data suggests that cAng1 specifically binds to N-cadherin on the myoblast surface. Moreover, satellite cells (PAX7^+^: red arrow in Supplementary Fig. 6a) and differentiating satellite cells (PAX7^−^: orange arrow-head in Supplementary Fig. 6a) on single fibers also expressed N-cadherin, which indicates that muscle lineage cells expressed N-cadherin (Supplementary Fig. 6a). N-cadherin expression on muscle stem cells was also observed during muscle regeneration after ischemic damage. The satellite cells (high N-cadherin signal, red arrow) and regenerating muscle fiber (weak N-cadherin signal, yellow arrow-head) expressed N-cadherin but not mature muscle fiber (white circle) (Supplementary Fig. 6b). We confirmed the binding between N-cadherin and cAng1 by an *in vitro* binding assay. cAng1 directly bound to recombinant human N-cadherin-Fc, and this interaction was significantly reduced with EGTA treatment (Fig. 4d), indicating that cAng1 binds to the surface of myoblasts through direct binding with N-cadherin in a Ca^2+^-dependent manner, activating p38MAPK downstream and myogenin expression.

### cAng1 activates N-cadherin by promoting the interaction between N-cadherin and p120-catenin

**Figure 5:**
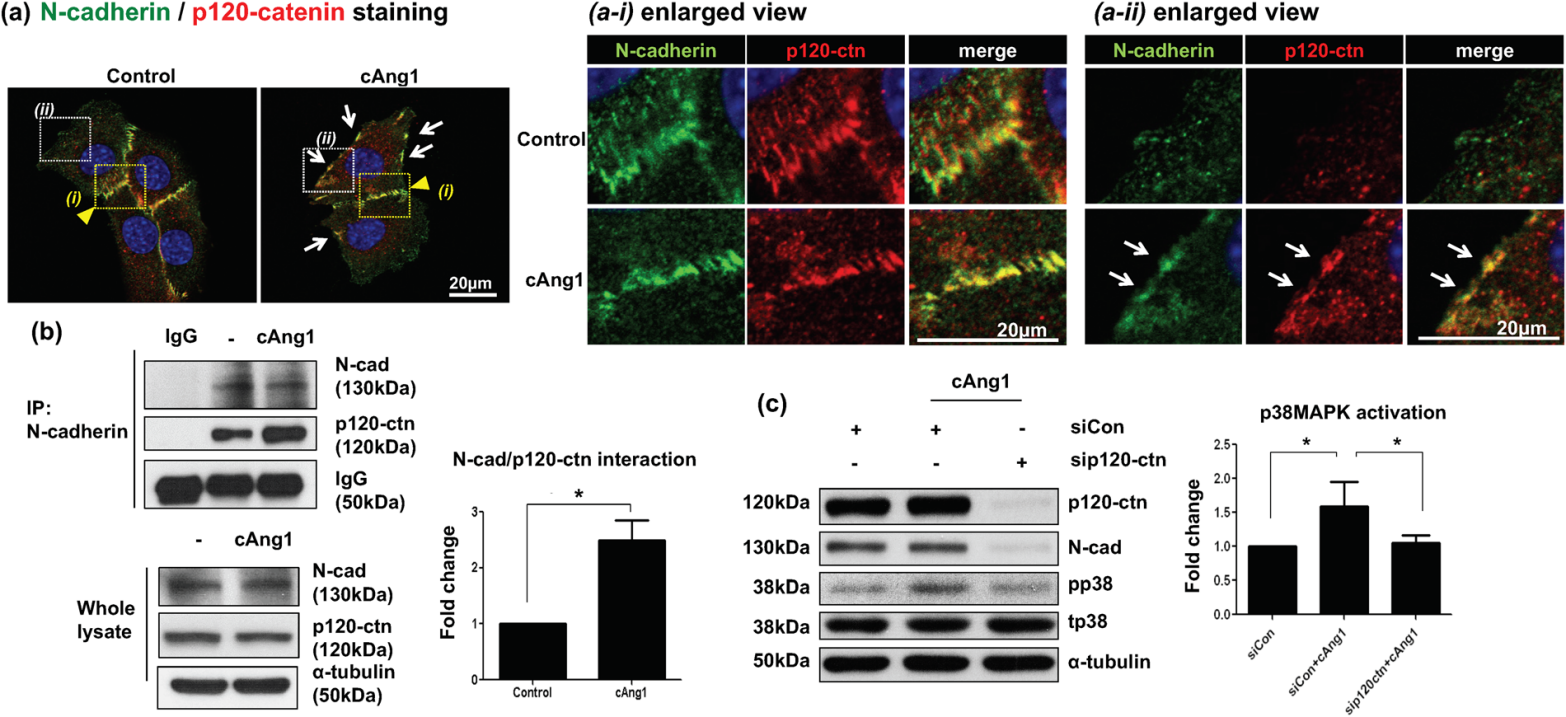
cAng1 activates N-cadherin by enhancing the formation of N-cadherin/p120-catenin complex. (**a**) Immunofluorescent staining for N-cadherin and p120-catenin. At the cell junction site (yellow arrowhead, a-i enlarged view), N-cadherin (green) and p120-catenin (p120-ctn, red) were co-localized regardless of cAng1 presence. At the non-junctional cell periphery (white arrows, a-ii enlarged view), however, co-localization of N-cadherin and p120-catenin was observed only in the presence of cAng1. Cell nucleus was indicated in blue by DAPI staining. Scale bar = 20 μm. Magnification, x630. (**b**) Immunoprecipitation analysis of N-cadherin and p120-catenin. cAng1 stimulated the association of N-cadherin with p120-catenin without affecting their protein amount. Quantitative graph of p120-catenin (Mean value ± SEM, n = 3, *P < 0.05). (**c**) Immunoblotting after p120-catenin depletion with siRNA. The siRNA against p120-catenin was transfected into myoblasts and the cells were threated under differentiation conditions. p120-catenin depletion reduced N-cadherin protein levels and inhibited p38MAPK phosphorylation at 3 hours in the presence of cAng1. The graph of p38MAPK phosphorylation ratio was assessed with densitometry of different immunoblots (Mean value ± SEM, n = 3, *P < 0.05).

It was reported that the formation of cell-cell junctions are induced by an N-cadherin homophilic interaction, while inactive N-cadherin interacts with β-catenin in cells without junctions^28^. The binding of p120-catenin to the cytosolic domain of N-cadherin induces adhesive dimerization, which forms a strong cell junction and induces adhesion signaling^28^. Therefore, we examined the effects of cAng1 on N-cadherin binding to p120-catenin (Fig. 5). We found that N-cadherin accumulated at the cell junction area (green) where p120-catenin (red) completely colocalized with N-cadherin (Fig. 5a-i). There was no difference in their colocalization at the cell-cell junction between control and cAng1-stimulated groups (Fig. 5a-i). By contrast, at the non-junctional cell periphery, the interaction between N-cadherin and p120-catenin was not observed in the control group, while the N-cadherin/p120-catenin interaction was remarkably enhanced by cAng1 stimulation (Fig. 5a-ii). Co-immunoprecipitation analysis confirmed that cAng1 induced the association of N-cadherin with p120-catenin without affecting their protein amounts (Fig. 5b). Of note, p120-catenin depletion by siRNA reduced N-cadherin protein as reported^29^ and inhibited p38MAPK phosphorylation even in the presence of cAng1 (Fig. 5c). These results indicate that the formation of the N-cadherin/p120-catenin complex is required for p38MAPK activation for cAng1-induced myoblast differentiation.

### cAng1 enhanced muscle regeneration after ischemic injury through myogenin expression

**Figure 6:**
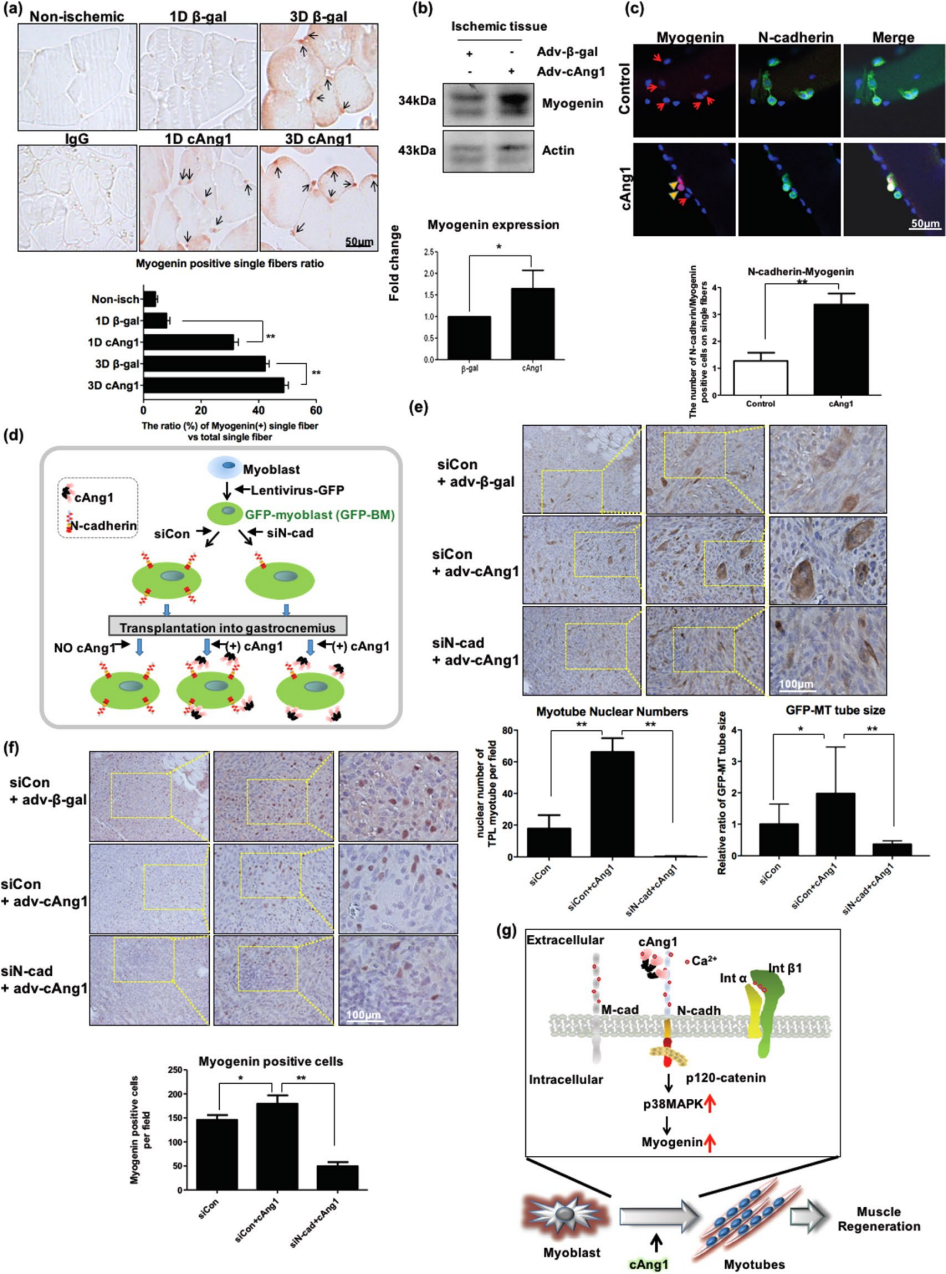
N-cadherin is important in myogenin expression in ischemic muscle and the cAng1-induced myotube formation of transplanted myoblasts. (**a**) Immunohistochemistry for myogenin in muscles at 1 and 3 days. Myogenin-expressing single fibers were identified with brown (myogenin) nuclei in muscle. Scale bar = 50 μm, Magnification, x400. Myogenin positivity was evaluated by counting the myogenin signal on single fibers and the ratio was assessed by normalization with total myofiber numbers in the field (0.47 mm × 0.63 mm). (n = 3, **P < 0.01). (**b**) Immunoblot analysis for myogenin in ischemic muscle at 1 day. Myogenin positive single fibers were increased by Adv-cAng1 compared to control. The quantitative graph of myogenin positive single fibers (n = 3, *P < 0.05). (**c**) The immunofluorescent staining of N-cadherin and myogenin on *ex vivo* single fibers. The single fibers were treated with differentiation media with or without cAng1 for 2 days. cAng1 enhanced myogenin expression of N-cadherin^+^ satellite cells on single fibers. The quantitative graph of N-cadherin-myogenin (n = 5, *P < 0.05). (**d**) Schematic diagram of myoblast transplantation experiment with N-cadherin knockdown. The GFP-expressing myoblasts (GFP-MB) were transfected with siRNA against N-cadherin or siControl. Each GFP-MB was transplanted into the gastrocnemius muscle of a nude mouse and then Adv-cAng1 or Adv-β-gal was injected. (**e**) The transplanted myoblasts were confirmed with anti-GFP antibody (brown) by immunohistochemistry. GFP-expressing myotubes (GFP-MT) generated from GFP-MB in mouse muscles were strongly stained as brown. The myotube generation in cAng1-treated groups was increased compared to control groups. However, N-cadherin knockdown myoblasts poorly generated GFP-MT. Scale bar = 100 μm, Magnification, x200. The nuclear numbers and the size of myotubes were analyzed in field (0.47 mm × 0.63 mm) (n = 3, **P < 0.01). (**f**) Immunohistochemistry for myogenin. Myogenin positive cells (red) were increased in the cAng1 treated group compared with control. In contrast, myogenin positive cells were dramatically reduced in the transplanted group of N-cadherin knockdown GFP-MB. Scale bar = 100 μm, Magnification, x200. The myogenin positive cells were counted on field (0.47 mm × 0.63 mm) (n = 3, *P < 0.05 and **P < 0.01). (**g**) Proposed model for myoblast differentiation and muscle regeneration through the cAng1/N-cadherin/p120-catenin/p38MAPK axis.

To verify the *in vitro* findings in *in vivo*, we examined whether cAng1 regulates myogenin expression after ischemic injury. Adv-β-gal or Adv-cAng1 was injected into the gastrocnemius muscle after femoral artery ligation (Fig. 6a,b). There was an upregulation of myogenin-positive satellite cells (arrows) with Adv-cAng1 injection compared to Adv-β-gal injection (Fig. 6a) as well as myogenin protein expression (Fig. 6b) in the ischemic limb one day after surgery. Of note, myogenin-expressing cells were rarely detected in sham-operated muscles (Fig. 6a). On day 3 after surgery, the Adv-β-gal injected group also increased myogenin positive single fibers. However, the Adv-cAng1 injected group displayed an increase in myogenin positive single fibers compared to the Adv-β-gal injected group (Supplementary Fig. 7). Moreover, cAng1 also increased myogenin (Fig. 6c) and Ki67 expression (Supplementary Fig. 8) in *ex vivo* satellite cells on single fibers under differentiation conditions. These data suggest that cAng1 enhances myoblast differentiation and muscle regeneration in ischemic muscles through upregulation of myogenic transcription factors such as myogenin.

### cAng1 accelerates myogenesis through N-cadherin in muscles

We found that N-cadherin is an important factor for cAng1-induced myoblast differentiation. We further examined whether cAng1 directly activated N-cadherin in myoblasts to promote myogenesis *in vivo*. The GFP-expressing myoblasts (GFP-MB) were transfected with siRNA against N-cadherin (siN-cad) or siControl (siCon). Each GFP-MB was transplanted into the gastrocnemius muscle of a nude mouse, followed by Adv-cAng1 or Adv-β-gal injection (Fig. 6d). Seven days after adenovirus injection, we examined whether the transplanted myoblasts formed myotubes by analyzing the GFP expression using an anti-GFP antibody (Fig. 6e and Supplementary Fig. 9).

In the siCon-treated group, transplanted GFP-MB (brown) formed a cluster in the gastrocnemius muscle. The numbers of GFP-expressing myotubes (GFP-MT), which have multi nuclear cells and analyzed with nucleus number and tube size of each GFP-MT to evaluate myogenesis, were markedly increased in the Adv-cAng1 injected group compared to muscles injected with Adv-β-gal. However, the cAng1-induced differentiation of GFP-MB to GFP-MT was inhibited in transplanted ‘siN-cad’-treated GFP-MB (Fig. 6e). Furthermore, the number of myogenin positive cells in muscles transplanted with siCon-treated GFP-MB was significantly increased by Adv-cAng1 injection compared to Adv-β-gal (Fig. 6f). The number of myogenin positive cells was decreased in the ‘siN-cad’-treated GFP-MB transplanted group even with Adv-cAng1 injection (Fig. 6f). These results suggest that N-cadherin has a central role in cAng1-induced myogenin expression and myotube generation.

## Discussion

Ang1 is known to induce angiogenesis through its interaction with Tie2 on endothelial cells^7^. Its primary function is the stabilization of vascular tubes and reducing vascular leakage^30^. Previous reports suggest that endogenous Ang1 from myoblasts induces myogenesis^18,19^. We investigated the endogenous effects of Ang1 on myoblast differentiation using siRNA and found that Ang1, but not Ang2, knockdown reduced myogenesis (Supplementary Fig. 10). Moreover, we examined the effects of native Ang1 and cAng1 on myogenesis. In agreement with previous reports, native Ang1 can induce myogenesis. However, its effect was weaker than cAng1 (Supplementary Fig. 2). The native monomeric Ang1 has to form multimeric structures to cluster the receptors, resulting in maximal signaling. cAng1 is already a pentameric structure, which is the reason why cAng1 has a stronger effect than native mono/multimeric Ang1^30,31^. Thus, we suggest that cAng1 is more suitable to clinical applications than native Ang1.

Ang1 was previously reported to bind integrin β1/3 in the presence of Ca^2+^ and to enhance survival signaling such as AKT/ERK in myoblasts and cardiomyocytes ‘under proliferating conditions’^16^. We also found that cAng1 enhanced cardiomyocyte survival through integrin β1 ‘under apoptotic conditions’ in our previous study^11^. Interestingly, we found that cAng1 did not activate AKT/ERK in skeletal myoblasts ‘under differentiation conditions’ at early time points (Supplementary Fig. 3). cAng1 could bind to myoblasts even with integrin β1 knocked down (Fig. 4c). These results suggest that cAng1-integrin β1 signaling in myoblasts might not be involved in ‘myoblast differentiation and myogenesis’.

AKT is also involved in myogenesis and Ang1 could enhance AKT signaling in myoblast differentiation^16,17^. However, phosphorylation of AKT S473 was increased after differentiation in both cAng1 and control groups (Supplementary Fig. 3). On the other hand, phosphorylation of p38MAPK was increased by cAng1 stimulation compared to control (Supplementary Fig. 3). Ivelisse Gonzalez *et al*. showed the relationship between p38MAPK and AKT during myogenesis. The early activation of p38MAPK signaling promotes AKT activation and enhances myogenesis, suggesting it is an important initiating factor in myogenesis^32^. In our study, cAng1 induced p38MAPK activation in early time points compared to control (Fig. 3a) and cAng1-induced myogenin expression was completely inhibited by a p38MAPK inhibitor (Fig. 3b), indicating cAng1 promoted myogenesis through the activation of p38MAPK signaling. Furthermore, when we introduced the Tie2 gene into myoblasts and stimulated them with cAng1, AKT was phosphorylated and activated without induction of myogenin because p38MAPK was not activated (Supplementary Fig. 4). These results demonstrate that cAng1 enhances myogenesis by inducing myogenin expression through p38MAPK activation.

Structurally, both native Ang1 and cAng1 contain the fibrinogen like domain on their C-terminal ends^5^. The fibrinogen like domain has numerous Ca^2+^ binding sites^16^. We found that Ca^2+^ ions have an important role in cAng1-induced myogenesis because Ca^2+^ ion chelation suppressed cAng1-induced p38MAPK activation and myogenin expression (Fig. 3b). Several groups reported that myogenesis is dependent on Ca^2+^ ions though signaling mechanisms such as Ca^2+^ ion intake and calmodulin-dependent kinase activity^33,34^. In contrast, we found that binding of cAng1 to myoblast surfaces relied on the presence of extracellular Ca^2+^ ions (Fig. 3c,d). These results suggest that cAng1 could activate p38MAPK signaling via binding on cell surface with extracellular Ca^2+^ ions.

Since myoblasts do not express Tie2, we focused on the cadherin and integrin families of receptors which have numerous Ca^2+^ binding domains with their activity reported to be regulated by calcium as well^16,27^. Interestingly, N-cadherin, M-cadherin, and integrin β1 are the three most expressed adhesion molecules on myoblasts, while the remaining adhesion molecules are not expressed or expressed at low levels (Fig. 4). Among these three adhesion molecules, specific knockdown of N-cadherin, but not M-cadherin or integrin β1, blocked cAng1-mediated p38MAPK activation. Moreover, cAng1 directly bound with N-cadherin in a Ca^2+^-dependent manner, which promotes the N-cadherin/p120-catenin interaction (Figs 4 and 5). These results suggest that formation of the cAng1/N-cadherin complex might be similar to the formation of the N-cadherin homophilic complex. Homophilic N-cadherin activation is regulated by Ca^2+^ ions and p120-catenin binding^27,29^, leading to p38 MAPK activation^35^. Another model may be proposed based on the structure of cAng1 and the N-cadherin complex. cAng1 is known to form pentamers and induce the clustering and activation of target molecules, such as the Tie1 endothelial-specific receptor tyrosine kinase and the Tie2 receptor^26,30,31,36^. Thus, when cAng1 binds to N-cadherin on the myoblast surface, cAng1 might cause N-cadherin clustering and structural changes, inducing the interaction with p120-catenin. We suggest that N-cadherin clustering by cAng1 is an important first step for the enhancement of myogenesis.

Abou-Khalil *et al*. reported that the Tie2 receptor is a positive regulator for the quiescent state muscle stem cell through the Ang1/ERK signaling pathway^8^. However, the Tie2 receptor does not exist in myoblasts, and we found that N-cadherin acts as a binding receptor for cAng1 and exists in muscle satellite cells (Supplementary Fig. 6). When we transfected Tie2 with overexpression plasmids to myoblasts, cAng1 activated the AKT pathway but suppressed p38MAPK activation as well as myogenin expression in differentiation conditions (Supplementary Fig. 4). Moreover, cAng1 enhanced satellite cell differentiation and proliferation in *ex vivo* single fiber culture (Fig. 6c and Supplementary Fig. 8) in differentiation conditions, which indicates that cAng1 regulates muscle differentiation and proliferation as previously reported^17^. Quiescent satellite or muscle stem cells express the Tie2 receptor which allow them to develop into myoblasts and this expression is lost in myoblasts^8^. We hypothesize that cAng1/Ang1-Tie2 signaling in myoblasts could suppress myogenesis by hindering intracellular signaling, such as p38MAPK activation, or by suppressing the cAng1/N-cadherin interaction due to the greater affinity of cAng1/Ang1 to the Tie2 receptor compared to N-cadherin.

We confirmed the importance of cAng1/N-cadherin signaling using the transplantation of GFP-expressing myoblasts into nude mouse gastrocnemius muscle (Fig. 6 and Supplementary Fig. 9). Transplanted myoblasts efficiently expressed myogenin and differentiated toward myotubes with cAng1 addition in mice. The myogenin expression in damaged muscle with cardiotoxin was detected after 3 and 7 days^37^ that also found in our system (Fig. 6f). However, when N-cadherin expression was suppressed by siRNA, cAng1 lost its effect in promoting myogenesis in myoblasts. Therefore, N-cadherin signaling in myoblasts is important in determining the fate of myogenic cells. On the contrary, Charlton *et al*. reported that the N-cadherin knockout mouse is embryonic lethal and N-cadherin null primary myoblasts from G418 selection on N-cadherin heterozygous primary myoblasts can form myotubes^38^. In our study, we also found that continuous knockdown of N-cadherin in myoblasts by lentivirus based short hairpin RNA (shRNA) reduced N-cadherin expression but did not reduce myogenin expression or myotube generation. Interestingly, we found that M-cadherin expression increased to a greater extent in N-cadherin shRNA knockdown myoblasts compared to control knockdown myoblasts (data not shown). This cadherin switch could compensate for the loss of N-cadherin because increased M-cadherin expression has also been shown to mediate myogenesis^39,40^. In our conditions, N-cadherin knockdown with siRNA in myoblasts did not induce M-cadherin expression (Fig. 4b). Thus, the level or time of N-cadherin knockdown might determine M-cadherin induction. Taken together, we suggest that N-cadherin signaling is an important factor to promote cAng1-induced myogenesis (Fig. 6g). Therefore, the cAng1/N-cadherin axis could be a suitable target for therapeutic applications to regenerate damaged muscles such as in ischemic injury.

## Materials and Methods

### Animal experiments and immunohistochemical staining

All experiments were approved by the Institutional Animal Care and Use Committee in Seoul National University Hospital (SNUH-IACUC) and animals were maintained in the facility accredited AAALAC International (#001169) in accordance with Guide for the Care and Use of Laboratory Animals 8th edition, NRC (2010) and the approval experimental protocol was SNU-110407-1 from Seoul National University Hospital. Male C57/BL6 mice were anesthetized with a 1:1 mixture of Rompun (10 mg/ml) and Zoletil (30 mg/kg) by intraperitoneal injection. To generate muscle ischemia, we removed a left limb unilateral femoral artery as previously described^14^. Mice in cAng1 groups were intramuscularly injected with Adv-cAng1 (1 × 10^9^ pfu) after surgery and mice in control group were injected with Adv-β-gal. To assess the myogenic efficiency of transplanted cells, GFP-MB (1 × 10^6^ cells) were injected with PBS into the gastrocnemius muscle. After 3 days, Adv-β-gal or adv-cAng1 (1 × 10^9^ pfu) were injected into the gastrocnemius muscle of the GFP-MB transplanted mouse. The ischemic limb volume was measured with a vernier caliper. The ischemic hindlimb and the transplanted muscle were harvested after 7 days, and made frozen or paraffin block sample. For histological analysis, 6~8 µm-thick slices were stained with H&E or Masson’s trichrome (Sigma). We performed immunohistochemistry using a Vector labs RTU ABC kit. Deparaffinized sections were incubated with specific primary antibody; Anti-myogenin antibody (1:200, Santacruz) or Anti-GFP (1:200, Santacruz). Signals were detected with chromogene such as DAB and NovaRed (Vector). The slide was observed under a microscope (Olympus BX50).

### Cell culture, adenovirus and lentivirus manipulation

All *in vitro* experiments were performed in accordance with regulations in the Institutional Biosafety Committee (IBC) of the Seoul National University Hospital (Registration Number of LMO Research Facility, LML08-49, 2009). Mouse C2C12 myoblasts were cultured with DMEM (Invitrogen) containing 10% FBS (Lonza) and antibiotics (Invitrogen). For myoblast differentiation, the C2C12 myoblasts were changed to differentiation media (DMEM containing 2% FBS). Myofiber explants were generated from C57BL/6 mice. Gastrocnemius muscle underwent enzymatic digestion at 37 °C in DMEM with 0.1% collagenase type II (Invitrogen) solution. Bulk myofibers with associated satellite cells were purified using multiple rounds of trituration, sedimentation and washing. The single fibers were incubated with differentiation media (DMEM/Glutamax (Invitrogen) containing 2% horse serum and 0.5% chick embryo extract). HEK293A cells (Q.Bio) and HEK293T cells (ATCC) were cultured with DMEM containing 10% FBS and antibiotics for amplification of adenovirus and lentivirus. For adenovirus amplification, Adenovirus-β-gal (Adv-β-gal) or Adenovirus-cAng1 (Adv-cAng1) was infected to HEK293A cells, and purified using CsCl gradient ultracentrifuge^41^. To construct lentivirus, pLp1, pLp2, and pLp3 (Invitrogen) were transfected to HEK293T cells with the pLL3.7-GFP vector. After 2 days, the transfected supernatants were harvested, cell debris was removed with a 0.22 µm filter, and then the supernatants were concentrated with an ultra-centrifuge. To induce lentivirus, the concentrated lentivirus supernatants were added to C2C12 cells with polybrene (8 mg/ml; Sigma). The GFP-expressing myoblasts (GFP-MB) were sorted by BD FACS aria.

### Knockdown of specific target genes and chemical inhibitor treatment

To suppress N-cadherin, M-cadherin, and Integrin β1, 40 nM siRNA compound (Santacruz) was incubated with Metafecten pro (Biomax) in PBS for 15 min at room temperature. Their mixtures were treated to flouting C2C12 myoblasts (1 × 10^5^ cells/ml) and seeded on 35 mm dish. Target gene knockdown was confirmed after 48 hours.

Chemical inhibitors were used 10 mM SB203580 (A.G. Scientific) for p38MAPK inhibition, and 2 mM ethylene glycol tetraacetic acid (EGTA, Sigma) for calcium ion chelator. To confirm myogenin expression, the chemical inhibitor was removed after 4 hours because the chemical inhibitor could be harmful to myoblast survival overnight.

### Immunoblotting and *in vitro* binding assay

Cells were washed twice with cold PBS and harvested by scraping with RIPA buffer [50 mM Tris (pH8.0), 150 mM NaCl, 1% Triton X-100, 0.1% SDS, 0.5% Deoxycholic acid, 10 mM β-glycerophosphate with 0.1 M NaF, 1 mM Orthovanadate, and protease inhibitor cocktail (Roche)]. The gastrocnemius muscle without bone was ground on a mortar with liquid nitrogen and resuspended with RIPA buffer. Protein concentration was determined using a BCA protein assay kit (Pierce). *In vitro* binding assay, immunoprecipitation was performed with immunocruz (Santacruz). The immunocruz beads were blocked with 1% BSA in PBS overnight at 4 °C. Human N-cadherin-Fc (1 ug, R&D system) was incubated with blocked beads in Ca^2+^/Mg^2+^ PBS (153 µM CaCl_2_ and 179 µM MgCl_2_) for 1 hour at 4 °C with gentle rotation. cAng1 (200 ng/ml) and EGTA (70 µM) were treated for 1 hour at 4 °C and then the mixtures were washed several times with Ca^2+^/Mg^2+^ PBS. For western blot analysis, prepared proteins were separated on SDS-PAGE and transferred to a polyvinylidene fluoride (PVDF) membrane (Millipore). The membrane was blocked with 3% BSA (Sigma) and incubated with the indicated antibodies overnight at 4 °C. Signal was detected with ECL (Invitrogen) or ECL-prime (Amersham). Anti-α-tubulin (Oncogene), anti-Myogenin, anti-Myf5, anti-MyoD, anti-N-cadherin, anti-M-cadherin, anti-Integrin β1, anti-phospho-p38MAPK (Thr180/Tyr182), anti-total p38MAPK, anti-phospho-ERK (Thr202/Tyr204), anti-p120-catenin (Santacruz), anti-total AKT (BD translation), anti-phospho-AKT (Ser473), anti-total ERK (Cell signaling), and anti-MyHC (Sigma).

### Immunofluorescence staining

C2C12 cells (1 × 10^4^ cells/well) were culture on 8-well 15 μm-Slide (Ibidi Treat) and single fibers were incubated with or without cAng1. Cells and single fibers were fixed with 4% PFA (WAKO) at room temperature for 10 min, and blocked with 1% BSA containing 0.05% Triton X-100 (Sigma) for cells and 0.1% Triton X-100 for single fibers at room temperature for 1 hour. The fixed cells and single fibers were incubated with primary antibodies [anti-MyHC (1:100, Sigma), anti-Myogenin (1:50, Santacurz) and anti-N-cadherin (1:100, Santacurz)] overnight at 4 °C followed by anti-mouse-Alexa 555 and anti-rabbit-Alexa 488 antibody (1:1000, Invitrogen) for 45 min at room temperature. The nucleus was stained with DAPI (Sigma). The fluorescent images were obtained using a confocal microscope (Carl Zeiss LSM710).

### Non-permeable protein cross-linking by 3,3’-dithiobis[sulfosuccinimi-dylpropionate] (DTSSP) and Fluorescence-activated cell sorting (FACS) analysis

To evaluate the adhesion of cAng1 on C2C12 myoblasts surface, floating cells were incubated with cAng1 in differentiation media for 1 hour at 37 **°**C and were treated with 2 mM DTSSP (Pierce) for 2 hours on ice for cross-linking proteins on the cell surface. DTSSP activity was stopped with 20 mM Tris buffer (pH 7.4)^24,42^. For calcium chelating, cells were pretreated with 2 mM EGTA (Sigma). After cross-linking by DTSSP, cells were stained using an anti-Flag antibody (Santacruz), as cAng1 was tagged with flag on its N-terminal domain^5,30^. The cAng1 binding affinity to C2C12 myoblasts was analyzed with BD FACS Canto II and DACO Summit®.

### Recovery capacity evaluation of ischemic limb with footprint

To evaluate recovery foot force, ischemic foot and non-ischemic foot were applied with ink. The mouse was walked through a paper tunnel. The foot print results were scored (0 to 4). The footprint assessment was conducted at day 1, 3, and 7, and there were five mice in each group.

### Statistical analysis

Quantification of band intensity was analyzed using ImageJ (Fiji). The results were expressed as mean ± standard error of the mean (SEM). The differences between the groups were compared by the unpaired *t-test* or one-way analysis of variance (ANOVA), followed by post hoc analysis with the Bonferroni test. P values ≤ 0.05 were considered statistically significant.

## Electronic supplementary material

Supplementary Information
