# Supplementary material for: COMP-Angiopoietin-1 accelerates muscle regeneration through N-cadherin activation
Source: Sci Rep. 2018 Aug 17;8:12323. doi: 10.1038/s41598-018-30513-7 (PMC6098079; doi:10.1038/s41598-018-30513-7)

COMP-Angiopoietin-1 accelerates muscle regeneration through N-cadherin activation

Seock-Won Youn, PhD.[1], Hyun-Chae Lee, PhD.[1, 2], Sae-Won Lee, PhD.[2], Jaewon Lee, BS.[1, 2],Hyunduk Jang, PhD.[2], Eun Ju Lee, PhD.[2], *Hyo-Soo Kim, MD, PhD.[1,2]

[1]Center of Cell- & Bio-Therapy for Heart, Diabetes, and Cancer, Seoul National University Hospital, Seoul; [2]Department of Molecular Medicine and Biopharmaceutical Sciences, Graduate School of Convergence Science and Technology, Seoul National University, Korea;

S-W.Y. and H-C.L. contributed equally to this work.

Corresponding author:

Hyo-Soo Kim, MD, PhD.

Professor, Department of Internal Medicine, Seoul National University Hospital,

Director, Center of CBT, SNUHospital

101 Daehak-ro, Jongno-gu, Seoul 110-744, Korea.

Phone: 82-2-2072-2226; Fax: 82-2-766-8904;

E-mail: hyosoo@snu.ac.kr or usahyosoo@gmail.com

Supplementary Text

Supplementary method

Evaluation of cAng1 signaling in myoblasts in a time dependent manner and Tie2 overexpression

To evaluate cAng1 initial signaling, myoblasts were treated with differentiation media with different time with or without cAng1. To checks the effects of Tie2, control plasmid (pFlag) or Tie2 plasmid were transfected in myoblasts with Metafecten Pro (Biomax). After 2 days, cells were treated with differentiation media. The initial signal of cAng1 in myoblasts was analyzed by the activation of AKT, ERK, and p38MAPK at 3 hours. Myogenesis was evaluated with myogenin expression after differentiation for 24 hours.

The stability of N-cadherin after trypsin detachment with Ca^{2+} ion

To investigate N-cadherin stability during trypsinization, myoblasts were detached by trypsinization with or without 100mM CaCl2. The detached cells were stained with anti-N-cadherin to evaluate surface N-cadherin expression. Using FACS, the N-cadherin expression on the cell surface was analyzed.

Immunofluorescent staining of single fibers and ischemic muscles

After single fiber isolation, the single fiber was stained with anti-Pax7 (1:25, DSHB) and anti-N-cadherin (1:100, Santacruz). To investigate satellite cell proliferation, the isolated single fibers were incubated on differentiation media (DMEM/Glutamax

(Invitrogen) containing 2% horse serum and 0.5% chick embryo extract) for 2 days and the single fibers were stained with anti-Pax7 and anti-Ki67 (BD Biosciences). After 7 days ischemia with Adv-β-gal or Adv-cAng1, the gastrocnemius muscles were processed to frozen blocks with OCT and sectioned at 6~8 μm thickness. The section was stained with anti-N-cadherin. The nucleus was stained with DAPI. Confocal microscope (LSM710 Meta) was used to take images.

The effects of Ang1 and Ang2 knockdown on myogenesis

During myogenesis, the mRNA level of Ang1 and Ang2 were evaluated using quantitative PCR (qPCR). After treatment with differentiation media, cells were harvested with Trizol (Invitrogen) and total RNA isolated. cDNA of 2ug total RNA was generated with a reverse transcription kit (TAKARA). qPCR was performed with the ABI system. To check the effects of Ang1, siRNAs among Ang1, Ang2 and control were transfected in myoblasts with Metafecten Pro and then cells were treated with differentiation media for 2 days. The expression of myosin heavy chain was performed through immunofluorescent staining and immunoblot. The expression of myogenin mRNA was evaluated with qPCR.

Supplementary figure legends

Supplementary figure 1. cAng1 increased regenerating single fiber in ischemic damaged muscle.

The masson's trichrome stain of ischemic gastrocnemius muscle at 7 days. Yellow line indicates ischemic damaged area. Lower panel showed ischemic damaged muscle area

and muscle regeneration. Scale bar is 200μm.

Supplementary figure 2. cAng1 promoted myogenesis more than native Ang1.

C2C12 myoblasts were treated with differentiation media with cAng1 (200ng/ml) and nAng1 (200 and 400ng/ml). After 5 days, cells were fixed and stained with anti-MyHC (red). Nucleus was stained with DAPI (blue). The quantification graph of the nucleus numbers of MyHC expressing myotubes is shown (*, $p< 0.05$ and **, $p< 0.01$).

Supplementary figure 3. cAng1 promoted p38MAPK activation.

C2C12 myoblasts were treated with differentiation media with or without cAng1. Cells were harvested at the indicated times. cAng1 increased the phosphorylation of p38 MAPK at early time points but not AKT and ERK. The quantification graph of each signal ratio was shown (Mean value ± SEM, n=3, *, $p<0.05$).

Supplementary figure 4. Tie2 inhibited the myogenic effects of cAng1.

Myoblasts with Tie2 overexpressed were treated with differentiation media with or without cAng1. After 3 hours and 24 hours, the cells were harvested and analyzed for differentiation signal and myogenin expression (Mean value ± SEM, n=3, *, $p<0.05$ and ns = None signification).

Supplementary figure 5. Surface N-cadherin expression was stable after trypsinization.

FACS analysis of surface N-cadherin expression. After trypsinization with or without CaCl2, cells were stained with anti-N-cadherin. Black is control IgG and gray is N-

cadherin expression. The expression of N-cadherin on the cell surface did not change in both groups.

Supplementary figure 6. Stellate cells and regenerating muscles in ischemic muscles expressed N-cadherin.

(**a**) Immunofluorescence of N-cadherin and Pax7 on single fibers. The isolated single fibers were stained with anti-N-cadherin and anti-Pax7. Red arrows and yellow arrowheads indicated Pax7 positive and negative myogenic cells, respectively. (**b**) Immunofluorescence of N-cadherin in ischemic muscles with Adv-cAng1 at 7 days. N-cadherin was stained in satellite cells and regenerating single fibers. The magnified figure is of the red square area. Red arrows indicated satellite cells or myoblast, yellow arrowheads indicated regenerating single fibers, and white circles indicated preexistent single fibers. Nucleus was stained with DAPI.

Supplementary figure 7. The analysis of myogenin positive satellite cells on ischemic damaged muscle fiber.

The immunohistochemistry of myogenin in ischemic damaged single muscle fiber. OCT section (7μm) of ischemic gastrocnemius muscle at 1 and 3 day was used to stain myogenin. The represented image indicates myogenin positivity (0, 1, 2, 3, and 4) on a single fiber. (a) Total single fiber number on ischemic area. (b) The ratio (%) of single myogenin positive fibers. Single myogenin positive fibers were counted and were divided by total muscle fiber number (n=3). (c) Multiple myogenin positive fiber ratio. Multiple myogenin positive fibers were counted and were divided by total muscle fiber number (n=3). The quantitative graph of myogenin positive single fiber (n=3, *, p<

0.05)

Supplementary figure 8. Satellite cells proliferation under differentiation condition. The isolated single fibers were incubated with or without cAng1 on differentiation media for 2 days. The fiber was stained with anti-Pax7 and anti-Ki67 to evaluate satellite cell proliferation. The quantitative graph of double positive cells ($Pax7^{+}Ki67^{+}$) (n=3, *, $p< 0.05$)

Supplementary figure 9. The transplanted GFP-MB location in gastrocnemius muscle.

After transplantation of GFP-MB, the myogenesis was evaluated with anti-GFP stain. In mimic diagram, "E" is endogenous muscle fiber and "G" is GFP-MB. siCon-GFP-MB with Adv-cAng1 increased GFP positive tubes compared to siCon-GFP-MB with Adv-β-gal and siN-cad-GFP-MB with Adv-cAng1.

Supplementary figure 10. Knockdown of Ang1 reduced myogenesis.

(**a**) The mRNA level of Ang1 and Ang2 in myoblasts that were exposed to differentiation media. The mRNA level of Ang1 increased in a time dependent manner but not Ang2. The relative value was based on Ang2 expression at 0hr (Mean value ± SEM, n=3, **, $P<0.01$). (**b**) Myoblast differentiation was evaluated after transfection with siAng1 and siAng2. Five days after transfection, myogenesis was assessed with skeletal myosin heavy chain (MyHC, red) expression. MyHC expression was inhibited by siAng1 compared with siCon and siAng2. Nucleus was stained with DAPI (Blue). Scale bar=100μm. Magnificatoin, X200. (**c**) Immunoblotting of MyHC after

differentiation for 3 days. It was significantly reduced by siAng1 compared with siCon and siAng2. (**d**) mRNA of myogenin was evaluated after differentiation media change 1 day with qPCR. The relative value was based on siCon expression at 0hr (Mean value ± SEM, n=3, **, $P<0.01$).

Table 1. primer list

Primer name		Sequence	Size
18S RNA	Forward	5'-GTAACCCGTTGAACCCCATT-3'	151bp
	Reverse	5'-CCATCCAATCGGTAGTAGCG-3'	
Myogenin	Forward	5'-GAGCGCGATCTCCGCTACAGAGG-3'	380bp
	Reverse	5'-CTGGCTTGTGGCAGCCCAGG-3'	
N-cadherin	Forward	5'-GGTGGAGGAGAAGAAGACCAG-3'	72bp
	Reverse	5'-GGCATCAGGCTCCACAGT-3'	
M-cadherin	Forward	5'-CTTGGGTGCCACGGATGA-3'	160bp
	Reverse	5'-ATGCAGGCCCTCGGAGAC-3'	
E-cadherin	Forward	5'-CCCGGGACAACGTTTATTAC-3'	72bp
	Reverse	5'-GCTGGCTCAAGTCAAAGTCC-3'	
VE-cadherin	Forward	5'-TCAACGCATCTGTGCCAGAG-3'	116bp
	Reverse	5'-CACGATTTGGTACAAGACAGTG-3'	
Cadherin 11	Forward	5'-CACAGGATGGTGTGGTGAAG-3'	170bp
	Reverse	5'-AGGCTCATCGGCATCTTCTA-3'	
Integrin b1	Forward	5'-CTGATTGGCTGGAGGAATGT-3'	173bp
	Reverse	5'-TGAGCAATTGAAGGATAATCATAG-3'	
Integrin b2	Forward	5'-AGTTCGACTACCCATCCGTG-3'	161bp
	Reverse	5'-GTTGCTGGAGTCGTCAGACA-3'	
Integrin b3	Forward	5'-CTGTTACAATATGAAGAATG-3'	177bp
	Reverse	5'-TTTTCATCACATACTGTAGC-3'	
Integrin b4	Forward	5'-TGTGTTCCAGGTGTTTGAGC-3'	507bp
	Reverse	5'-TTTCTCATCATTGCGGTTCA-3'	
Angiopoietin-1	Forward	5'-CTCGTCAGACATTCATCATCCAG-3'	138bp
	Reverse	5'-CACCTTCTTTAGTGCAAAGGCT-3'	
Angiopoietin-2	Forward	5'-TCCAAGAGCTCGGTTGCTAT-3'	114bp
	Reverse	5'-AGTTGGGGAAGGTCAGTGTG-3'	

Supplementary figure 1.

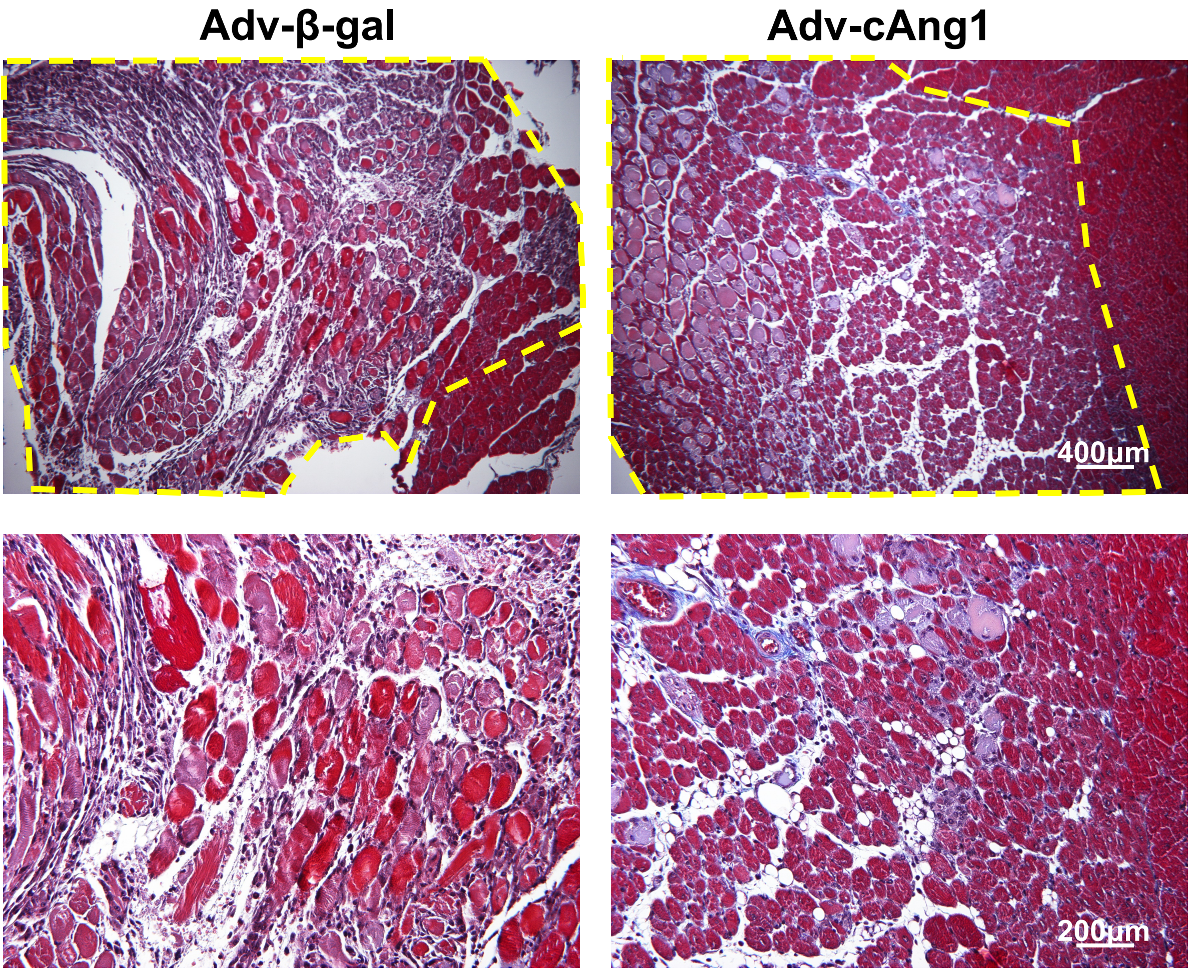

Supplementary figure 2.

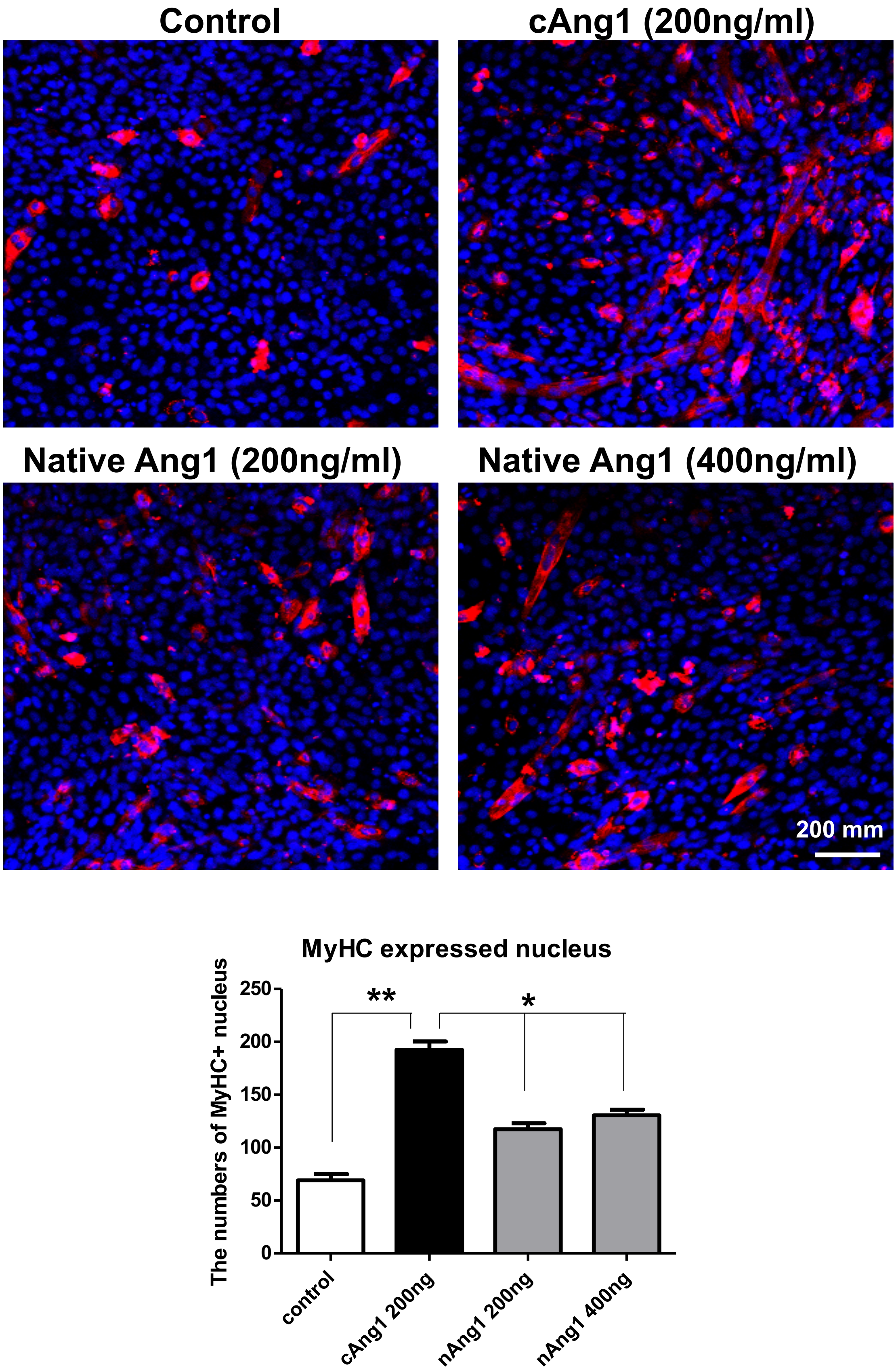

Supplementary figure 3.

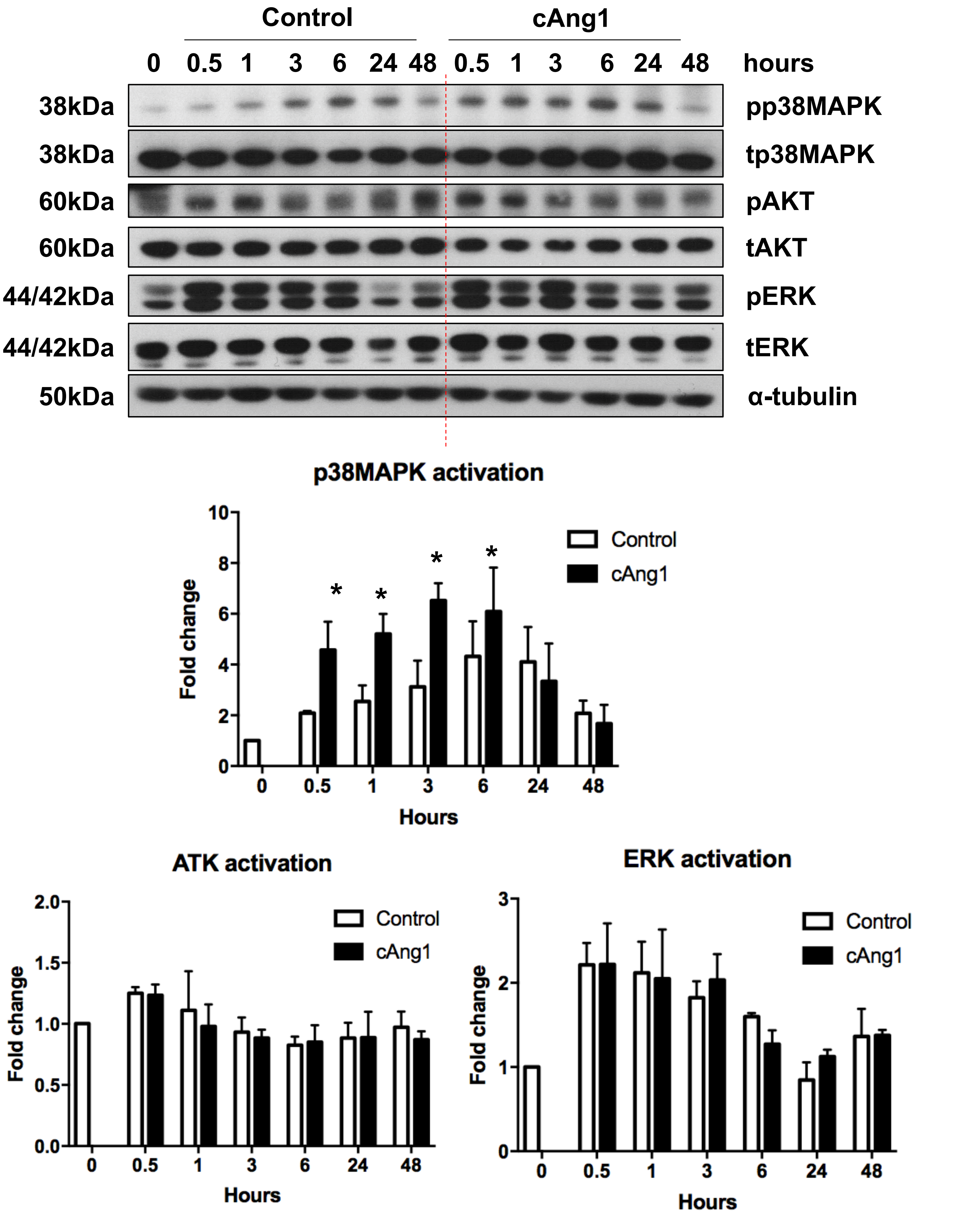

Supplementary figure 4.

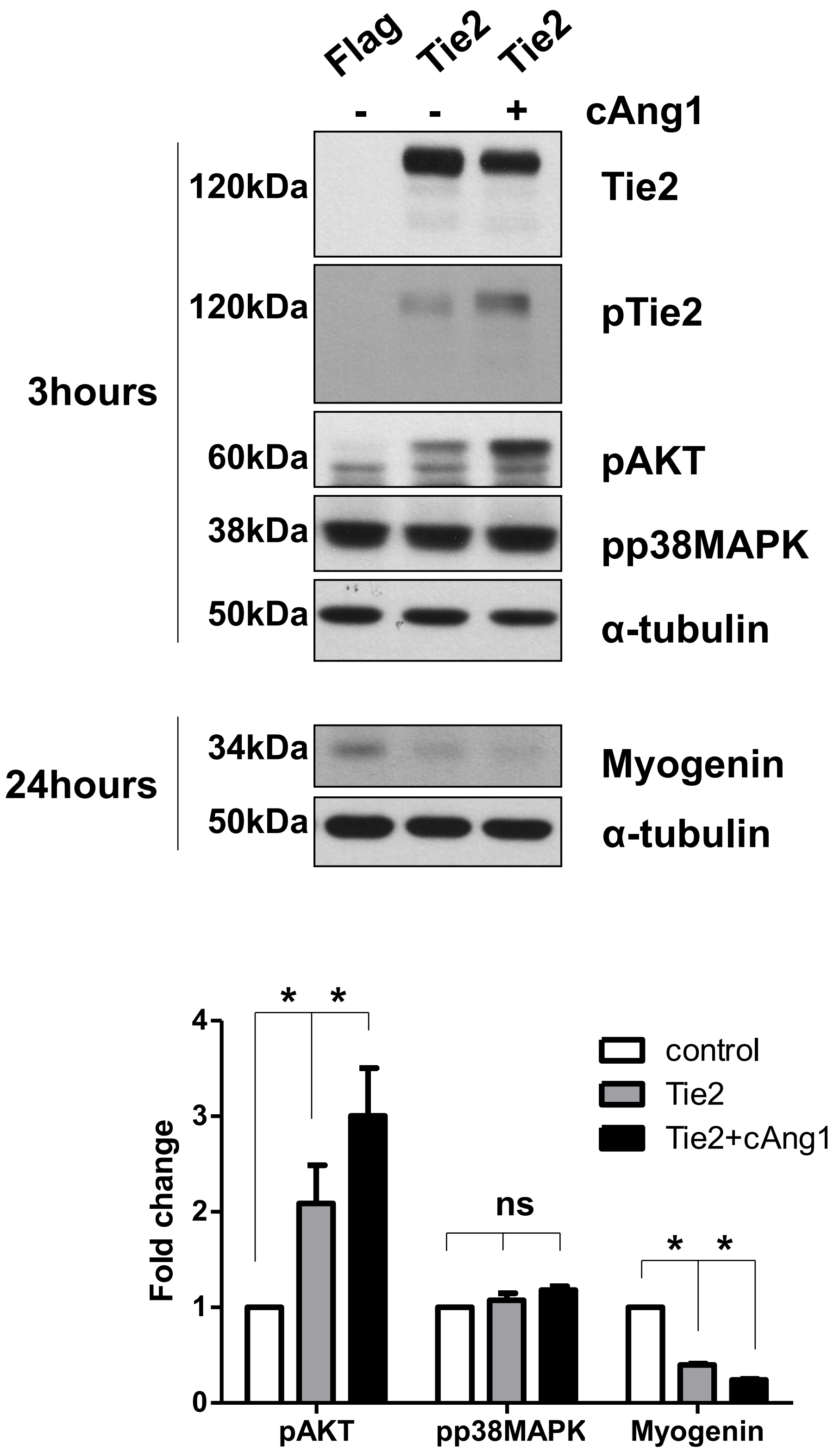

Supplementary figure 5.

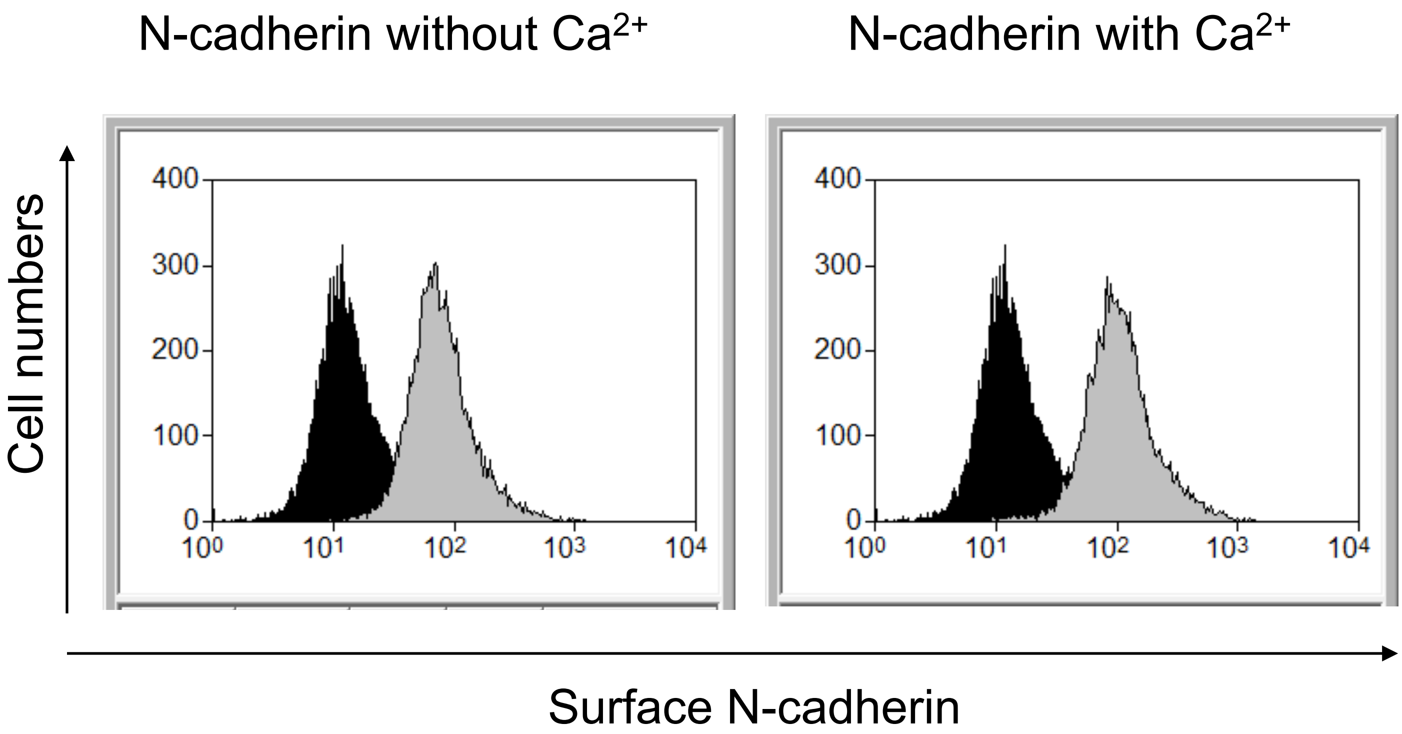

Supplementary figure 6.

(a)

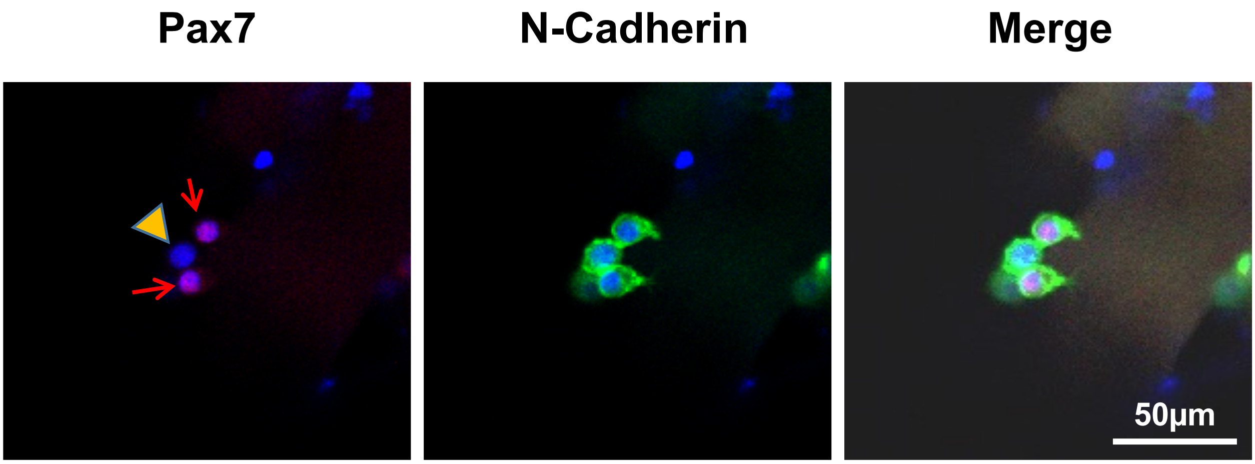

(b)

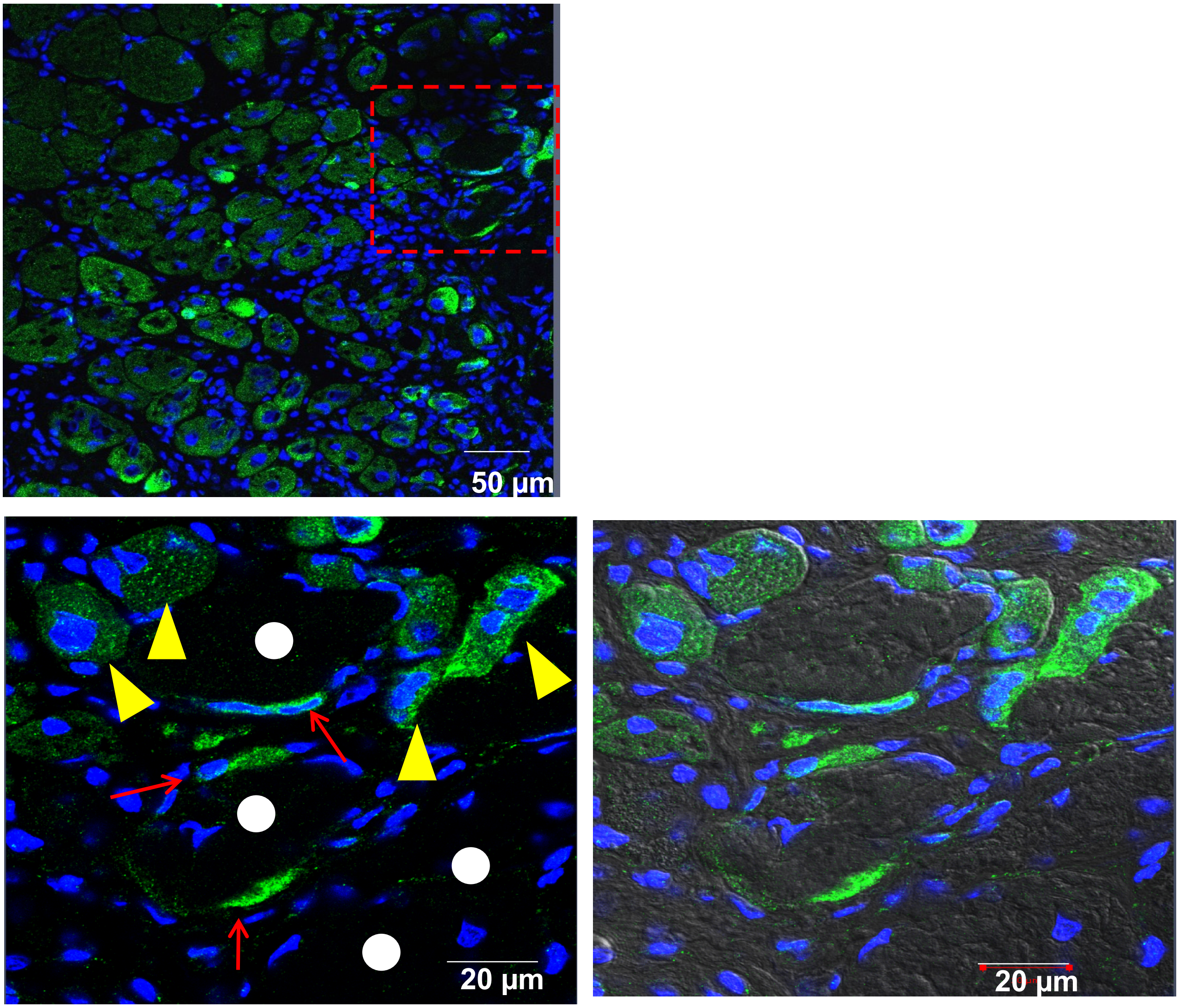

Supplement figure 7.

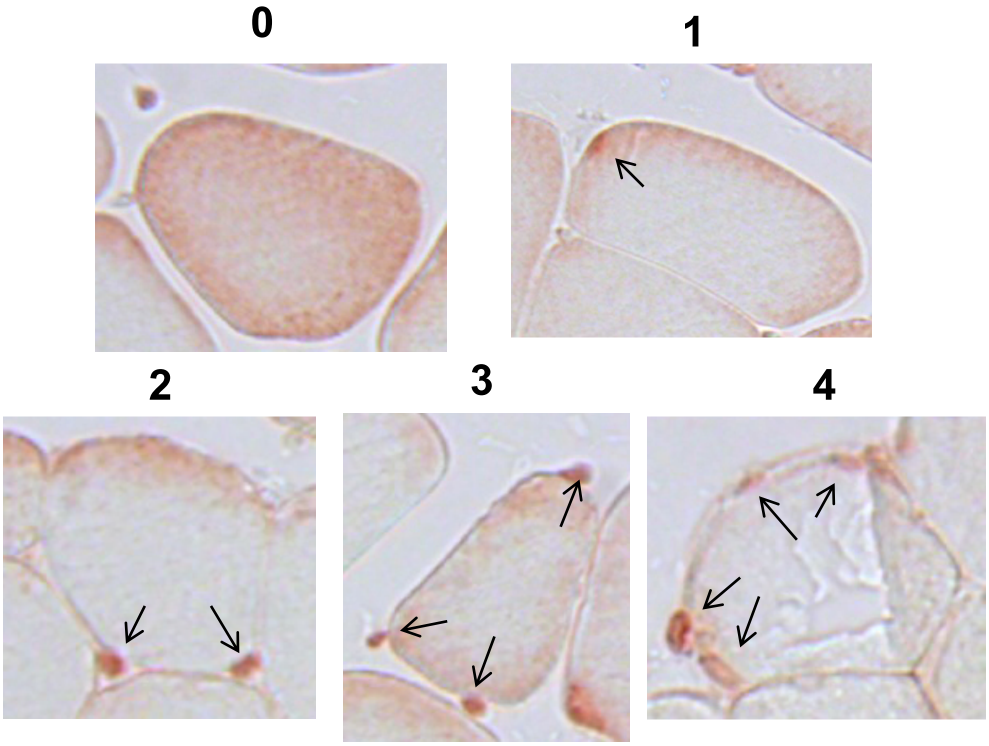

(a) Total single fiber number per field

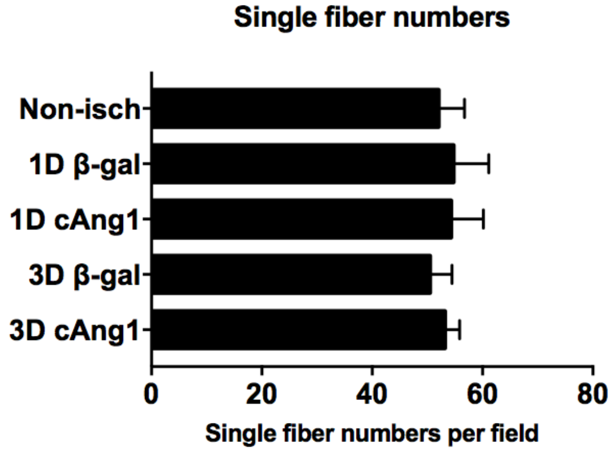

(b) Myogenin positive single fiber (one)

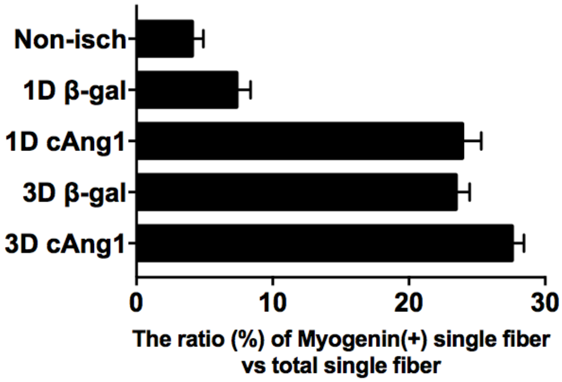

(c) Myogenin positive single fiber (Two more)

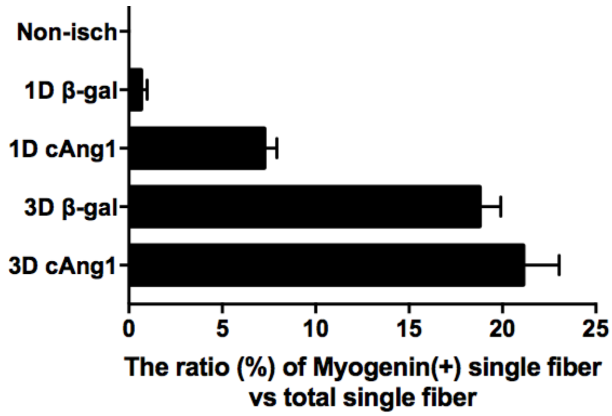

Supplementary figure 8.

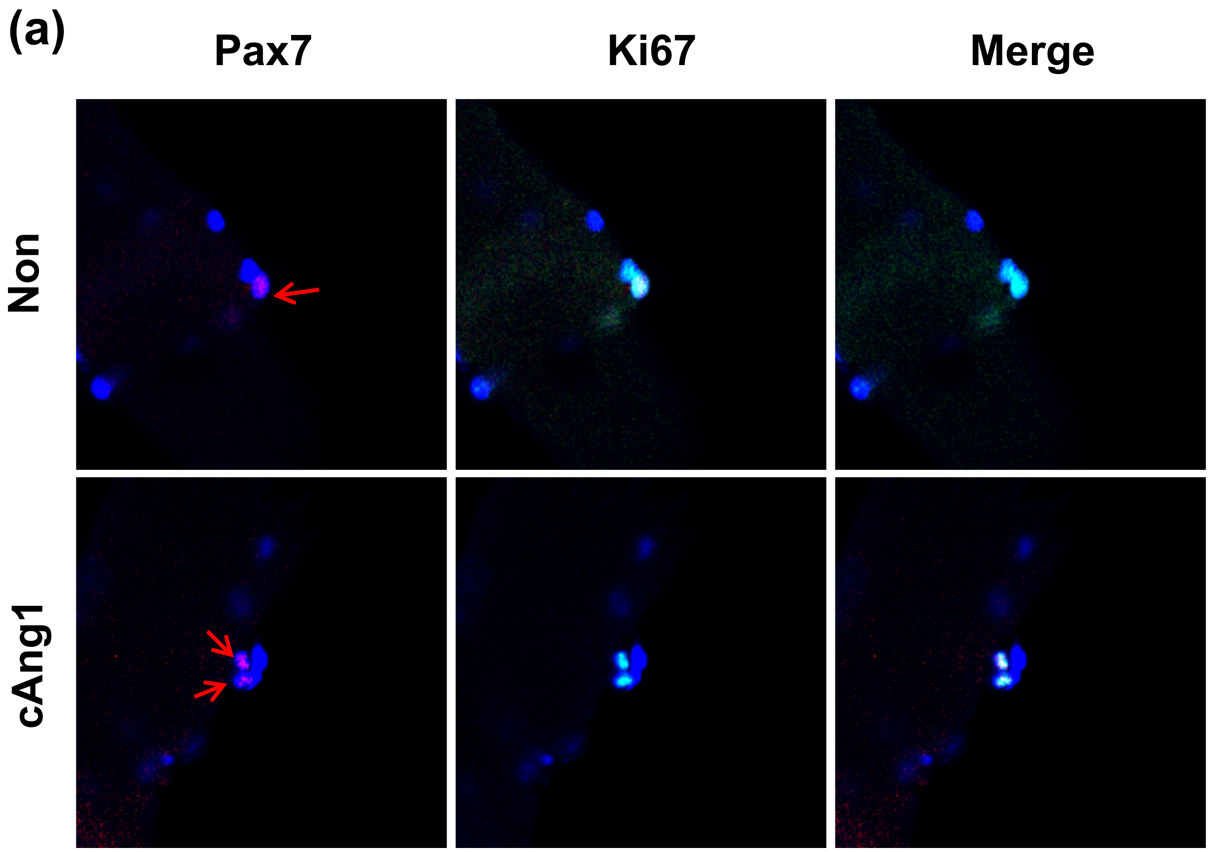

→ **Undifferentiation and proliferation satellite cells**

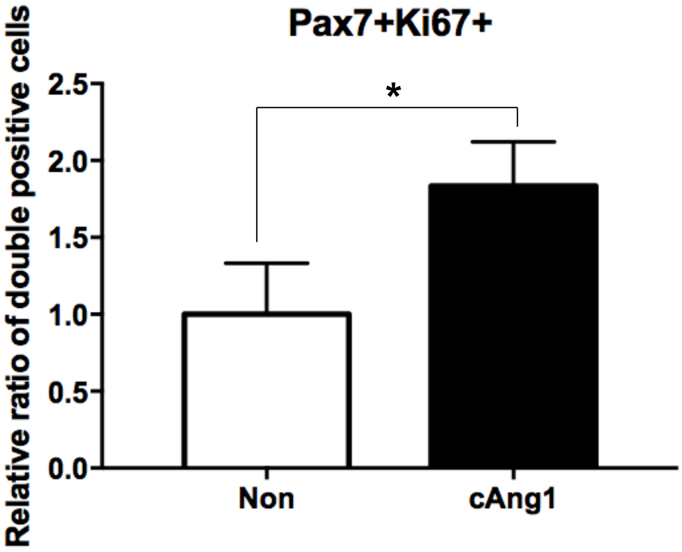

Supplement figure 9.

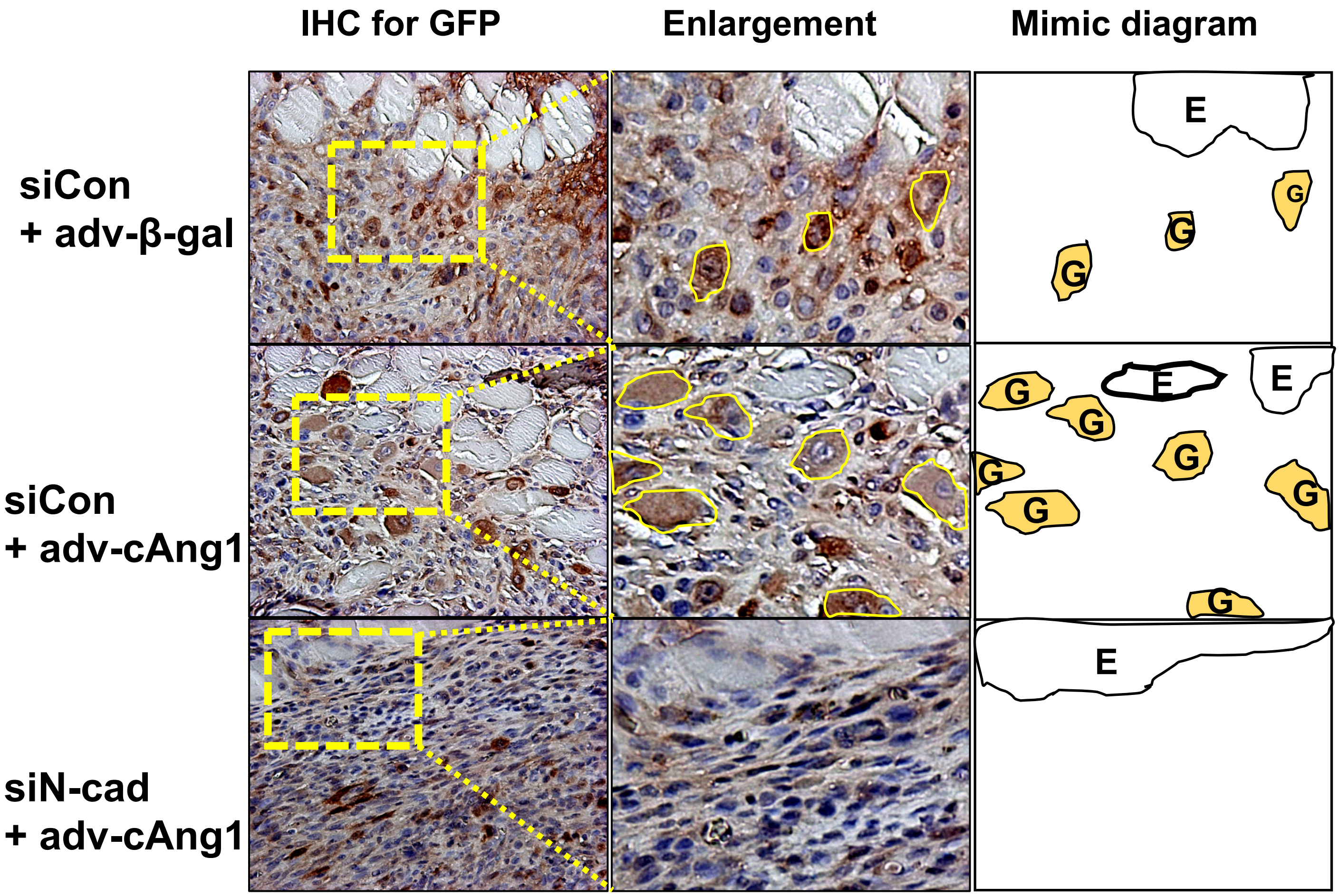

Supplementary figure 10.

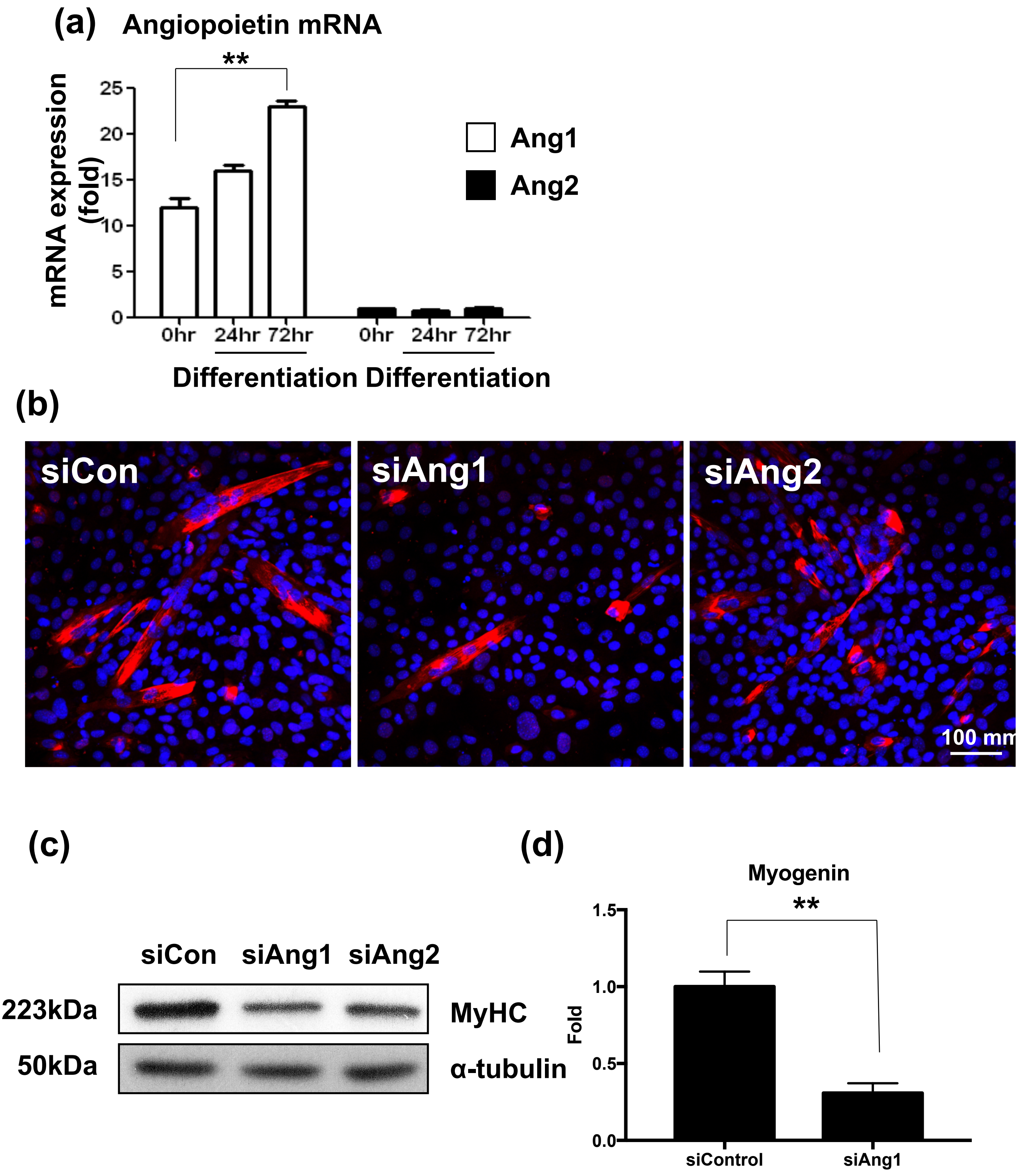

Supplement: Supplementary file 1 — Supplementary Information [file 41598_2018_30513_MOESM1_ESM.pdf]
